# Adsorptive, electrochemical and computational approaches for the recovery of precious metals from wastewater

**DOI:** 10.1039/d6ra00376a

**Published:** 2026-05-15

**Authors:** Ayesha Taj, Suniya Shahzad, Afzal Shah, Mustafa Tuzen, Abdul Haleem

**Affiliations:** a Department of Chemistry, Quaid-i-Azam University Islamabad 45320 Pakistan afzals_qau@yahoo.com; b Tokat Gaziosmanpaşa University, Faculty of Science and Arts, Chemistry Department 60250 Tokat Turkey; c Ningbo Institute of Digital Twin, Eastern Institute of Technology Ningbo Zhejiang 315200 China

## Abstract

The global shortage of clean water and rising demand for precious metals have intensified interest in alternative resource streams beyond conventional mining. Wastewater, particularly from electronic manufacturing and electroplating processes, often contains metal concentrations that exceed those found in natural ores. This characteristic positions wastewater not only as a pollutant matrix but also as a valuable secondary resource. With increasing demand for both water availability and metal supply chains, attention has gradually shifted toward alternative recovery routes that can serve dual purposes. In this regard, the present review takes a closer look at the combined use of adsorption and electrochemical techniques, not as parallel options, but as interdependent steps within a single process. Individually, each method brings certain strengths, although neither is without limitations. Adsorption is widely recognized for its ability to selectively capture and concentrate metal ions, even in fairly complex aqueous environments. Once metals are taken up, their regeneration and recovery often requiring additional chemicals and, in some cases, generating secondary waste streams that are challenging to manage. Electrochemical approaches, by contrast, allow direct metal recovery through reduction processes and can regenerate the working surface under suitable conditions. However, these systems tend to lose effectiveness when selectivity becomes critical, particularly in multi-ion solutions where competing species interfere. In this perspective, adsorption is integrated with electrochemical treatment. The adsorption step effectively isolates and enriches the target species while the electrochemical step of electroreduction and recovery facilitates regeneration without extensive chemical input. Though it is not a perfect solution in every case, yet its advantages cannot be overlooked. The current review brings these aspects together by linking material characteristics with process performance, examining a range of adsorbent systems with emphasis on how structural features influence selectivity and reuse, alongside the emerging role of computational approaches in the design of adsorbents and the optimization of conditions to enhance the uptake and recovery of precious metals. Overall, the integration of adsorption and electrochemical methods minimizes reagent usage and facilitates cyclic operations for the recovery of precious metals from wastewater, which is increasingly significant for promoting circular resource utilization.

## Abstract 2

The global shortage of clean water and rising demand for precious metals have intensified interest in alternative resource streams beyond conventional mining.

## Graphical abstract

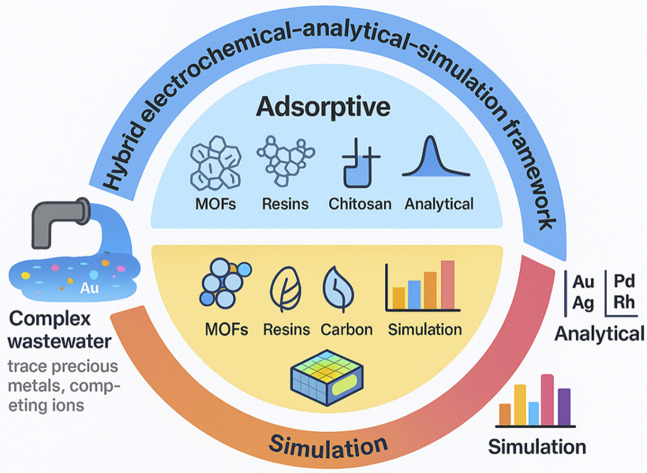

## 1. Introduction

The swift advancement of electronic industries, catalysis, and clean energy technologies has led to the demand for precious metals. Precious metals are defined by their rarity, economic value, and chemical properties, with eight rare transition elements including gold (Au), silver (Ag), platinum (Pt), palladium (Pd), iridium (Ir), rhodium (Rh), osmium (Os), and ruthenium (Ru). These metals have distinct variations in softness, hardness, melting points, and densities. Their exceptional resistance to oxidation and corrosion, combined with appealing electrical, thermal, and catalytic properties, has enabled their extensive use across various applications in daily life.^1^ Notably, palladium and rhodium serve as crucial catalysts in hydrogenation processes and in reducing emissions from automobiles, while gold and silver are vital for nanophotonic systems, healthcare diagnostics, and efficient sensors. The release of precious metals into wastewater stems from a diverse array of industrial, human, and process-related activities.^2,3^

Metal-contaminated drinking water and wastewater pose a significant threat to the environment. Hence, removing these metals is crucial, as their recovery offers both environmental protection and economic value due to their widespread industrial applications. Subpar recycling methods and ineffective leaching processes for waste electrical and electronic equipment (WEEE), the oil, gas, and petrochemical sectors are significant contributors, primarily due to their heavy reliance on platinum-group metals (PGMs) as catalysts have also become notable secondary sources of precious metals.^4^ Traditional mining and refining methods like hydrometallurgy and pyro-metallurgy are energy intensive, environmentally damaging, circumscribed by slow separation kinetics, poor economic feasibility, and generate considerable greenhouse gas emissions along with toxic waste.^5^

Wastewater valorization mitigates resource depletion and lowers the environmental damage. It supplements the natural ores satiating the global demand for precious metals. Certain industrial effluents particularly from electronic manufacturing and electroplating contain metal concentrations significantly higher than natural ores (*e.g.*, ∼200 g ton^−1^ Au in effluents compared to 5–30 g ton^−1^ in conventional ores), thereby positioning wastewater not only as a pollutant matrix but also as a valuable secondary resource.^6^ Separation and recovery of precious metals in their pure form become strenuous when their low concentrations are present in waste streams and secondary resources. However, the feasibility of precious metals recovery cannot be assessed solely on the concentration threshold. Although ultra trace levels such as <0.1 ppb may seem economically insignificant at first glance, recovery potential is governed by the combined effect of volumetric accretion, process integration, concentration, and metal market value. For instance, large-scale industrial facilities processing thousands of cubic meters of wastewater per day can concurrently discharge recoverable quantities of gold even at sub-ppb levels. Moreover, adsorption systems endorse selective pre-concentration of noble metals from dilute matrices, aptly enriching trace metals to recoverable levels before electrochemical reclamation.^7,8^

Although adsorbents serve as primary concentrators of precious metals, allowing for their selective capture and accumulation from diluted and multi-ion systems, electrochemical techniques also have a significant impact on the recovery and reuse process. Conventional electrochemical recovery rely on the direct reduction of dissolved metal ions onto electrode surfaces, where metal deposition occurs through electrochemical reactions such as electrodialysis, electrochemical reduction,^9^ electrocoagulation^10^ and electroosmosis.^11^ These processes are more effective for solutions containing relatively high metal concentrations. In contrast, adsorption–electrochemical hybrid processes integrate adsorption with electrochemical reduction, enabling efficient metal capture even from dilute wastewater streams. In this approach, metal ions are first adsorbed onto the surface of an adsorbent and after adsorption, an applied electrical potential induces the *in situ* electrochemical reduction of the adsorbed metal ions, converting them into metallic deposits or nanoparticles while simultaneously regenerating the adsorption sites.^12^

The electrochemically mediated adsorption strategy represents an emerging hybrid approach that integrates the selectivity of adsorption with the controllability of electrochemical processes for efficient recovery of metals. In this system, redox-active functionalized electrodes serve as both the capture medium and the separation interface. Upon application of an appropriate anodic potential, neutral redox centres are oxidized to positively charged sites, which selectively bind anionic metal complexes. This electrostatically driven adsorption is highly specific and rapid, occurring within minutes even in multicomponent solutions. Importantly, the process is reversible: switching the potential to a reductive regime neutralizes the binding sites, triggering the release of the adsorbed metal species into a concentrated stream. As a result, adsorption, desorption, and enrichment are achieved within a single operational unit, eliminating the need for external chemical regenerants and enabling cyclic reuse of the adsorbent.

Compared to conventional adsorption techniques such as activated carbon or ion-exchange resins, the electrochemical–adsorption approach offers distinct advantages in terms of selectivity and operational sustainability. Traditional adsorbents, although widely used, often suffer from limited specificity in the presence of competing ions and require chemically intensive regeneration steps involving acids, bases, or organic solvents. These additional treatments not only increase operational costs but also generate secondary waste streams. In contrast, electrochemically controlled adsorption relies solely on electrical input to modulate binding affinity, thereby minimizing chemical consumption and simplifying process design. Furthermore, the ability to precisely tune the applied potential allows for enhanced discrimination between metal species, leading to significantly improved separation performance.

When compared with purely electrochemical methods such as electrodeposition or capacitive deionization, the hybrid approach demonstrates superior functionality. Conventional electrochemical techniques are typically governed by redox potentials and mass transport limitations, which can result in poor selectivity and interference from co-existing metal ions. Electrodeposition, for instance, often leads to co-reduction of multiple species, while capacitive systems lack molecular-level specificity. The incorporation of redox-active adsorbent materials in electrochemical–adsorption systems address these limitations by introducing tailored binding interactions at the electrode surface. This enables selective capture of target metal complexes without relying solely on electrochemical reduction or physical ion storage mechanisms. In the reported study, a redox-active electrochemical–adsorption system was developed for the selective recovery of gold from complex aqueous matrices.^13^ The authors employed a poly(vinylferrocene)-functionalized carbon nanotube (PVF–CNT) electrode, where ferrocene moieties act as reversible redox centers. Upon application of an anodic potential, these centers are oxidized to positively charged ferrocenium species, which electrostatically attract and bind anionic gold complexes, particularly dicyanoaurate ions. The adsorption process was found to be rapid and highly selective, even in the presence of competing metal ions. Subsequent reversal of the applied potential reduces the ferrocenium sites back to their neutral state, resulting in controlled desorption and recovery of gold in a concentrated form. This cyclic capture–release mechanism enabled high adsorption capacity, near-complete recovery efficiency, and effective regeneration of the electrode without the use of chemical eluents.^13^

Electrosorption as a faradaic controlled process can overcome the limitations associated with dilute metal streams. The incorporation of redox-active interfaces with electrochemical driving forces improve ion selectivity and eliminate issues such as concentration polarization and slow mass transit, commonly faced in traditional electrochemical recovery systems. Thus, the amalgamation of adsorption and electrochemical modulation has been underscored as a crucial method to improve metals recovery approaches.^13^ Smith *et al.* also demonstrated significant increase in separation performance by electrochemically controlled adsorption in dilute metal systems by coupling faradaic reactions with surface binding processes. Their research showed that by applying potential the surface charge and binding affinity of redox-active interfaces can be changed, resulting in improved selectivity and reduced limitations such as slow mass transport and poor ion discrimination that are commonly observed in conventional electrochemical recovery methods.^14^ Lee *et al.* examined the electrocoagulation-assisted recovery of precious metals, where electrochemically generated hydroxide species that functioned as adsorptive sites for metal capture. This study indicated elevated removal efficiencies for Au and Ag, emphasizing that adsorption phenomena can be intrinsically incorporated within electrochemical systems. However, the selectivity of the proposed method was not as good as redox-mediated electrosorption because it used non-specific binding mechanisms.^15^ In contrast, Wang *et al.* explored advanced adsorbent materials such as carbon nanostructures and metal–organic frameworks for recovery of the precious metals through pure adsorption. These materials exhibited high adsorption capacities, and their regeneration typically requires chemical eluents and multiple processing steps, which increase the operational complexity and the environmental burden. The absence of an external control mechanism further limits their adaptability in multi-ion systems.^16^

Overall, the integration of adsorption and electrochemical control within a single platform provides a compelling pathway for process intensification in metals recovery. By combining high selectivity, rapid kinetics, and energy-efficient regeneration, electrochemical-adsorption systems offer a viable alternative to conventional technologies, particularly for the recovery of valuable metals from dilute and complex streams such as electronic waste and mining effluents. This synergistic mechanism not only improves mass transfer efficiency but also allows controlled recovery, selective separation, and cyclic reuse of adsorbents without the need for harsh chemical regeneration agents. Such hybrid approaches have gained a considerable attention for the recovery of valuable metals from industrial and mining wastewater. Computational tools elucidate interfacial interactions and reaction pathways to rationally optimize both material design and process performance, particularly DFT, molecular dynamics and finite-element modeling provide molecular-level insights into adsorption mechanisms and electrode design, thus accelerating rational material development and process optimization. This review emphasizes the interconnectedness and complementary nature of these three methods, presenting them as a cohesive framework rather than as separate technologies. This perspective is crucial for advancing sustainable wastewater treatment and enhancing resource recovery strategies.

## 2. Precious metals loaded wastewater effluents

Industrial wastewater rivulets vary widely in precious metals concentration, composition, and volume. These streams roll out from electroplating, catalyst production, photographic processing, electronics manufacturing, and several other sectors. Evaluated concentrations of precious metals across these streams provide backdrop for evaluating recovery feasibility. Subsequent sections present specific industries and processes that contribute to the incorporation of precious metals in waterbodies. Table 1 enlists the estimated concentration of precious metals in the effluents of these industries and processes.

**Table 1:** Estimated concentration of precious metals in various effluents

Source	Metal	Concentration range	References
e-Waste leachate	Au	>200 g ton^−1^	6
	Pd	1500–24 700 L^−1^	20 and 21
	Ag	3970 mg L^−1^	10
Electroplating industry	Ag	500–1000 mg L^−1^	22
Waste fixer solutions	Ag	5000–10 000 mg L^−1^	19
Jewelry manufacturing	Pd	4.7 mg kg^−1^	21
Cyanide leachate (mining)	Au	∼7.15 mg L^−1^	15
	Ag	∼305 mg L^−1^	
Industrial Ag wastewater	Ag	2–20 mg L^−1^ (ultra-low); >1000 mg L^−1^ (high)	23
Mining wastewater (field data)	Ag	∼0.8–1.1 mg L^−1^	24

### 2.1. Electroplating industry

Electroplating deposits heavy and noble metals such as Ni, Au, Ag, Cu, Pt, Cr, Ni, Fe and Zn on materials to improve their conductivity, corrosion resistance and wear resistance. In cleaning processes, these metals enter wastewater and generate electroplating sludge containing high concentration of metals.^17^

### 2.2. Electronic waste leachate

E-waste recycling processes generate leachates containing several metals due to the disintegration of printed circuit boards and electronic components. These leachates typically contain metals such as Pb, Cu, Fe, and trace precious metals including Ag, Au, and Pd depending on the leaching conditions. Hydrometallurgical processing of waste printed circuit boards generates leachates containing valuable metals such as Au, Ag, and Pd along with base metals. Such waste are considered as potential secondary resources for precious metals recovery.^18^

### 2.3. Water fixer solutions

Photographic processing generates effluent containing high amount of dissolved silver in the form of silver thiosulfate complexes. The significantly high silver concentration in these waste streams make them one of the richest secondary sources for silver recovery.^19^

### 2.4. Jewelry manufacturing wastewater

Wastewater generated during jewelry production processes such as polishing, cleaning and plating contains dissolved metals including Cu, Au, Rh, Ag, Ni, and Zn. Copper concentrations in jewelry wastewater reaches to approximately 200 mg L^−1^ along with trace precious metals.^25^

## 3. Mechanisms involved in adsorptive recovery of precious metals from wastewater

Adsorption is a process by which atoms, particles, or ions, collectively termed as adsorbates, assemble on the surface of a material, known as the adsorbent. It is primarily classified into physisorption, which enables multilayer adsorption by weak, nonspecific interactions, and chemisorption, which involves the formation of strong chemical bonds between the adsorbent and adsorbate owing to unbalanced surface forces on solids or liquid adsorbents, which attract and retain the adsorbate molecules at the interface. The adsorption of precious metal ions from aqueous solutions generally occurs through multiple mechanisms, including electrostatic attraction, ion exchange, surface complexation, coordination bonding, and redox interactions. The extent to which each mechanism contributes is influenced by parameters such as pH, ionic strength, the surface chemistry of the adsorbent, and the speciation of the metal ions.

### 3.1. Electrostatic interactions

**Fig. 1:**
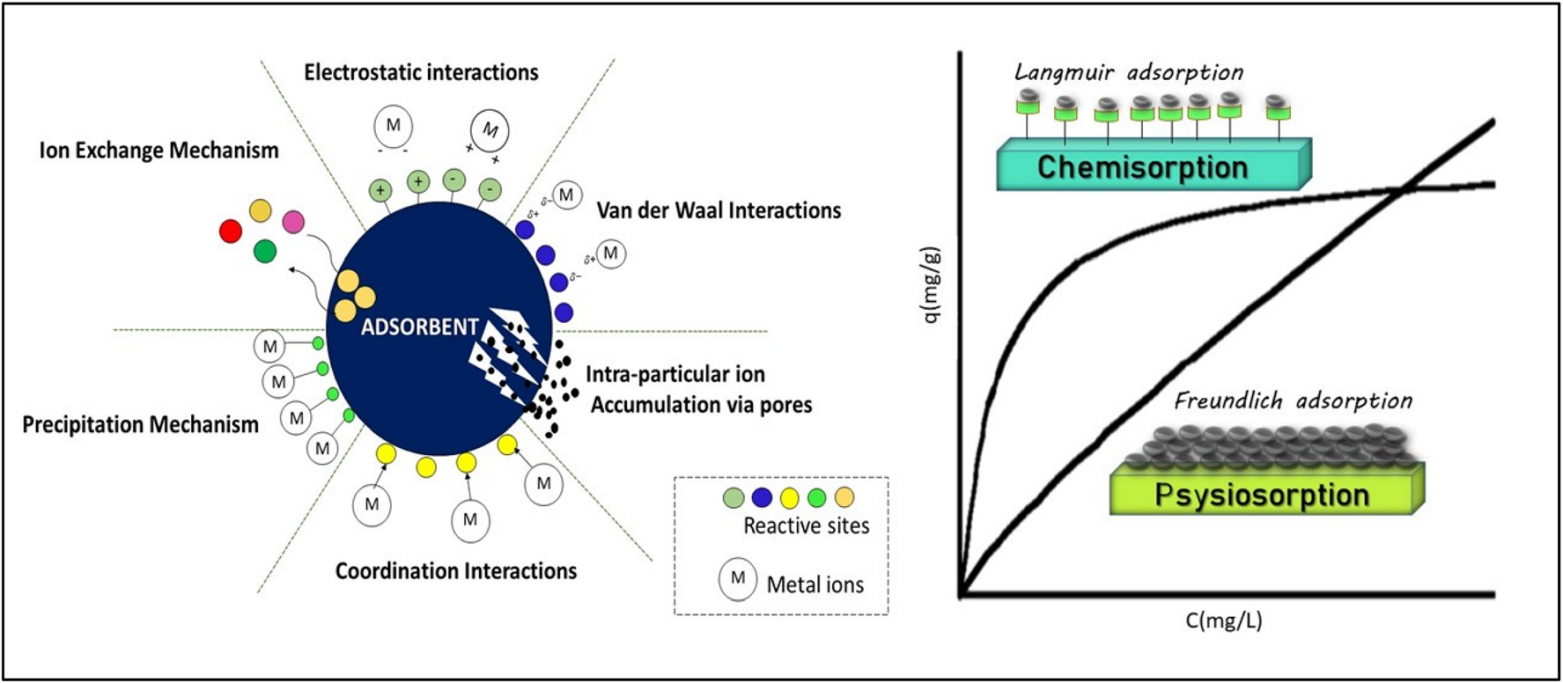
Schematic representation of mechanisms involved in adsorption process. The figure illustrates the mechanisms governing adsorption through ion exchange, electrostatic and van der Waals interactions, intra-particle ion accumulation through pores, coordination bonding, and precipitation. Different colors mark reactive sites on the adsorbent, and “M” signifies metal ions (left), illustration of the formation of monolayer and multilayers on the adsorbent surface following Langmuir and Freundlish isotherms (right).

One of the primary mechanisms governing adsorption of positively charged metals from solutions are electrostatic interactions. As negatively charged functional sites carboxylate (–COO^−^), hydroxyl (–OH^−^), sulfonate (–SO_3_^−^), phosphate (–PO_4_^3−^), phosphine, pyridine, and other heterocyclic groups, are commonly present on the adsorbent surface such as thiourea resins,^26^ activated carbon,^27^ chitosan,^28^ clay minerals, metal oxides, and biosorbents.^29^ In acidic medium, the protonation of functional groups occurs which reduces the negative charge density on the surface of adsorbent thus lowering the electrostatic attractions, whereas in basic media, deprotonation intensify the negative charge density, thus encouraging stronger adsorption.^30^ Ions such as, K^+^, Na^+^ or Ca^2+^ may compete with the same binding sites where target metal ion is supposed to be adsorbed thus reducing the adsorption efficiency. Fig. 1 represents the involvement of redox reactions, van der Waals interactions and ion exchange processes often complementing the electrostatic interactions to increase adsorption efficiency.

### 3.2. Ion exchange mechanism

Ion exchange is a mechanism which is commonly exhibited by zeolites, ion exchange resins,^31^ clay minerals,^32^ functionalized polymers, that have negatively charged surface sites or other replaceable ions which can be substituted owing to their high electron affinity values. This process is highly influenced by hydration energy, ionic radius, and charge density. Larger the ionic radius means greater polarizability, stronger is the binding to exchangeable sites, highly selective is the ion exchange.

### 3.3. Surface complexations

Surface complexation or coordination interactions involves the formation of chemical bonds between functional groups such as sulphur, nitrogen or oxygen containing ligands *e.g.* thiol (–SH), amine (–NH_2_), hydroxyl (–OH), carboxyl (–COOH) and target metal ions forming a coordination complex. The basic mechanism is the sharing of pair of electrons between functional groups on the surface of adsorbent and the metal ions. Hard Soft Acid Base (HSAB) concept is pivotal for soft acid soft base interactions. For instance, Silver ions have greater affinity for thiol ligands.^33^ The presence of competing ions may interfere complexation mechanism as they struggle for the same binding site where target metal adsorbs thus reducing the adsorption efficiency. This mechanism is common in materials that have active surface groups capable of chelation or coordination such MOFs,^34^ covalent organic frameworks (COFs),^35^ Chitosan^28^ and other polymers.

### 3.4. Redox mechanism

Redox mechanism direct mobility, chemical state of ions thus influencing the removal efficiency in adsorption mechanism. These reactions are primarily involved in reduction of metal cations to their elemental form in the presence of electron rich functional groups on the surface of adsorbent or any reducing agents.^36^ This is commonly observed in biomass derived adsorbents and graphene oxide (GO) which encourage ion immobilization *via* precipitation.

## 4. Types of adsorbents used for precious metals recovery

### 4.1. Clay minerals

Clay minerals such as kaolinite, montmorillonite, and bentonite are among the earliest adsorbents used for water purification due to their natural abundance, low cost, and high cation exchange capacity. Their layered negatively charged surfaces enable the adsorption of various metal ions through ion exchange and surface complexation mechanisms. Yap *et al.* incorporated aminoclay into a magnetic graphene–Fe_3_O_4_ nanocomposite, achieving rapid adsorption of the ions of Au, Ag, and Pd from waste streams.^32^ Mosai *et al.* investigated the recovery of Ir, Pd, and Rh ions by bentonite when it was functionalized with 3-aminopropyl(diethoxy)methylsilane, thus proving it as a potential adsorbent for efficient recovery of metal ions from mining and industrial wastewaters.^37^ Nevertheless, natural clay minerals typically exhibit lower adsorption capacities compared with engineered adsorbents such as nanoparticles or MOFs.

### 4.2. Activated carbon & carbon-based materials

Traditionally, activated carbon has been used as a commercial adsorbent material for Au(CN)_2_^−^ complexes since the 1970s, however, recent advances in the development of hybrid and graphene-based nanocomposites have inspired the design and fabrication of materials such as graphene (G), carbon nanotubes (CNTs), reduced graphene oxide (rGO) graphene oxide (GO),^38^ and other carbon based sorbents. They have gained attention due to their exceptional sorption capacities, extraordinary efficiencies, tunable surface chemistry, and remarkable electrical conductivity, making them ideal candidates for the removal of precious metals from waste water.

Reilly *et al.* synthesized a thiol-functionalized activated carbon through the formation of peptide bonds with cysteamine thus enabling the attachment of –SH groups through covalent bonds. It achieved up to 100% Au recovery within 8 hours and facilitated the formation of Au(iii) in acidic medium, whereas basic environments promoted both Au^0^ nucleation, suggesting coordination and reduction were involved in the process.^27^ Magnetic modification of activated carbon (MAC) by the integration of superparamagnetic properties achieved an outstanding efficiency of 99.1% from cyanide solution within 5 hours.^36^ Birtane *et al.* developed a CNT cellulose nanocomposite synthesized *via* thiol–ene reaction that can selectively adsorb gold ions even in complex systems such as river water mainly due to the successful incorporation of sulphur bearing functional groups which plays a vital role in gold coordination.^39^ An advancement was made by the fabrication of a multivariate biological metal when combined with organic framework (MTV-BioMOF), thiol-functionalized single-walled carbon nanotube buckypapers (MTV-BioMOF@HS-SWCNT-BP), reveals thiol and thioether functional groups within the MOF framework where the CNTs facilitate electron transfer. The material exhibits up to 98% selectivity towards gold with a maximum adsorption efficiency of 600 mg g^−1^.^40^ Gold adsorption by a new class of polyethylene glycol/graphene/carbon nanotube (PEG/G/CNT) nanocomposites was studied by Khan *et al.* used an ultrasonically assisted dissolution process, manipulating mixed graphene and CNT loadings within polyethyleneglycol PEG polymer matrix. The material exhibits remarkable affinity towards Au(iii) ions among several tested ions such as Cu^2+^, Pb^2+^, Y^3+^, Cr^3+^, Zn^2+^, Co^2+^, and Fe^3+^. The maximum adsorption capacity of PEG/G/CNT for Au(iii) reached 80.80 mg g^−1^, which was further substantiated by ICP-OES analysis, thus highlighting the importance of CNT-based composites as an efficient material for precious metal recovery.^41^ Graphene oxide, owing to its large surface area and oxygenated functional groups, has appeared as an effective adsorbent for gold recovery from leaching systems. The interactions occur through surface complexation and electrostatic interactions rather than redox reactions. The adsorption studies of gold glycinate complex (Au(gly)_2_) on GO demonstrated an uptake efficiency of 7.4 mg g^−1^, the loading capacity remains lower than that of sulfur-containing adsorbents.^38^

Silver containing systems such as ammonia-free thiosulfate solutions when treated with powdered activated carbon (PAC) demonstrated an effective adsorption of achieving over 92% recovery across a wide pH range governed by surface complexation.^42^ Meanwhile, sulfhydryl-functionalized activated carbon (AC-SH-80) displayed an adsorption efficiency up to 719.2 mg g^−1^, acting as a superior selective adsorbent, capable of efficiently capturing Ag^+^ even from complex wastewater matrices, attributed to its electrostatic chelation with C–O/C=O functionalities, and reduction of Ag^+^ to metallic Ag^0^*via* S–H groups.^33^ Conversely, Fan *et al.* developed S-doped porous carbons (SPCs) derived from passion fruit (*Passiflora Edulis* Sims) shells where the sulphur acted as an active site, significantly improving the Ag^+^ adsorption performance up to 115 mg g^−1^ by the formation of Ag_2_S nanoparticles.^43^ In contrast supermagnetic biochar/polyurea formaldehyde nanocomposite (SMCsr-B/PUF) coupled bio-derived carbonaceous functionality and spinel CoFe_2_O_4_ magnetism, promoting rapid microwave-assisted adsorption of silver quantum dots (Ag-QDs) exhibiting >93% removal within 15–25 seconds. The main driving forces were surface adsorption–coagulation mechanism complemented by electrostatic and π–π interactions between Ag-QDs and biochar.^44^ Heshami *et al.* established that glycinate complexes of Au and Ag bear strong attraction to graphene oxide than with pristine graphene, confirming the role of oxygenated functionalities in charge transfer and surface affinity.^45^ The comparatively higher adsorption energies of glycinates on graphene oxide, compared to cyanide complexes, indicated that non-cyanide ligands such as glycinate can achieve high adsorption. GSH/Fe_3_O_4_@GO nanocomposite introduced by Zounia *et al.* developed a dual-function architecture with glutathione-derived thiol and carboxyl sites where sulfur, oxygen, and nitrogen functionalities contribute to high adsorption capacity (586 mg g^−1^) of Ag–CN complex.^46^ Similarly, gallic acid-modified GO (GO/GA) owing to polyphenolic hydroxyl groups facilitated Ag^+^ binding primarily through electrostatic and complexation interactions at neutral pH. The system demonstrated 98.35% silver removal under mild conditions within 60 minutes.^47^ A strategic advancement beyond conventional adsorption-based systems was made by the integration of photo thermal COF architectures such as (ethidium-based covalent organic framework decorated on carbon nanotubes) EB-COF@CNTs, which induce charge migration by coupling solar energy conversion with selective metal ion capture. The conjugation of electron-rich cationic COFs with conductive carbon nanotubes produced a synergistic interface that not only widened the light-absorption range but also enhanced electron mobility, overcoming the optical and transport limitations of pristine COFs. The resulting 277.01 mg g^−1^ silver ion uptake confirmed that photo-responsive materials can achieve comparable or superior efficiency to chemically functionalized adsorbents through solar-driven activation rather than chemical reduction.^35^

Palladium recovery by bamboo stem activated carbon (BSAC), demonstrates a sustainable and cost-effective approach from man-made electroless plating (ELP) solutions. The mechanistic insights revealed multiple physicochemical interactions due to presence of oxygenated functional groups (–OH, –COOH, and C=O) on the BSAC surface that serves as electron-donating sites, thus enable complexation and electrostatic attraction with Pd(ii) species. The synergistic use of cationic surfactant (CTAB) modulates surface charge and hydrophobicity, thereby enhancing Pd(ii) binding *via* micelle-mediated adsorption and ion-pair formation. It achieves superior Pd(ii) uptake (6.69–50.13 mg g^−1^) and 80% removal efficiency, outperforming conventional agitation methods.^48^ Chen *et al.* developed a three dimensional reduced graphene oxide (rGO) aerogel functionalized with sulfamic acid that introduce –SO_3_H and –NH functionalities to enhance electrostatic attraction and hydrogen bonding with cationic Pd(ii) species under alkaline conditions. Ethylenediamine provided abundant nitrogen and sulfur functionalities, that contributed in chelating –NH_2_ sites thus facilitating bidentate complex formation *via* N → Pd(ii) coordination. This results in a remarkable 97.0% adsorption efficiency within 50 minutes.^49^ However, in glycine–cyanide leach systems, glycine acts as both a complexing and buffering agent, coordinates with Pd(ii) through its amine and carboxylate groups, on the other hand, cyanide enhances leaching efficiency by forming [Pd(CN)_4_]^2−^ complexes. The adsorption of these species onto activated carbon is governed by π–π electron interactions between Pd–cyanide complexes and the graphitic planes, along with secondary electrostatic and van der Waals forces. Optimal adsorption at pH 9.5 reflects the favorable surface charge compatibility between deprotonated carbon sites and the anionic Pd complexes resulting in 98.2% Pd recovery due to valence electron exchange and surface complexation.^50^ Khan *et al.* fabricated Ti_3_AlC_2_ MAX phase graphene oxide (GO) nanocomposite by combining the thermal conductivity, intrinsic stability, and eco-friendliness of Ti_3_AlC_2_ with porosity enriched surface and abundant oxygenated chemical moieties of GO which provides abundant adsorption sites, enhancing Pd(ii) uptake through chelation and also facilitate Pd ion reduction, thereby enhancing both selectivity and adsorption capacity.^51^

### 4.3. Silica gels

Silica gel is used as inorganic adsorbent owing to its large surface area, highly porous structure, and abundant surface silanol groups. These functional groups (Si–OH) enable strong adsorption interactions with metal ions through electrostatic attraction, hydrogen bonding, and complexation mechanisms. Researches have shown that functionalized silica-based materials can achieve selective recovery of precious metals from aqueous solutions. For instance, Mphahlele *et al.* report >90% removal of Pt/Pd by designing two silica supported adsorbents, an acylthiourea-bearing gel (DTMSP-BT-SG) and an amine-bearing gel (BTMSPA-SG). They concluded that DTMSP-BT-SG achieved higher Pt and Pd recovery with faster kinetics. In a follow-up study, the same group reported that optimizing the amine-functionalized silica (APTES-SG) can dramatically improve its affinity towards Pd/Pt up to 97%, thus emphasizing the impact of synthesis on performance.^52,53^ Conversely, Herman *et al.* incorporated (3-mercaptopropyl)-silane (a thiol) into porous silica and attained high loading capacity up to 238 mg g^−1^ across pH 4–9 thus, highlighting the potential of silica-based materials in advanced wastewater treatment technologies.^54^ However, despite encouraging adsorption attributes, silica gels manifest lower adsorption capacities with some metal ions in complex industrial wastewater containing competing species. Furthermore, regeneration of silica gel adsorbents requires chemical treatments that may reduce structural stability after repeated cycles.

### 4.4. Activated alumina (AA)

Activated alumina (γ-Al_2_O_3_) is high-surface area inorganic adsorbent especially for oxyanions. It has an amphoteric oxide surface *i.e.*, at pH below its point of zero charge (∼8.1), the surface is protonated for attracting anions *via* ligand exchange, however, above that pH it deprotonates and can bind cations. Mechanistically, alumina removes metals through a combination of hydroxyl exchange and surface complexation. Adsorption by activated alumina relies on its network of micro and meso pores with high density of –OH sites.^55^ Regarding cost and scalability, AA is very inexpensive and can be regenerated simply by acid or base washes, recovering anion capacity. Therefore, AA is tailored for the recovery of anion-forming precious metals complexes.

### 4.5. Ion-exchange resins

**Fig. 2:**
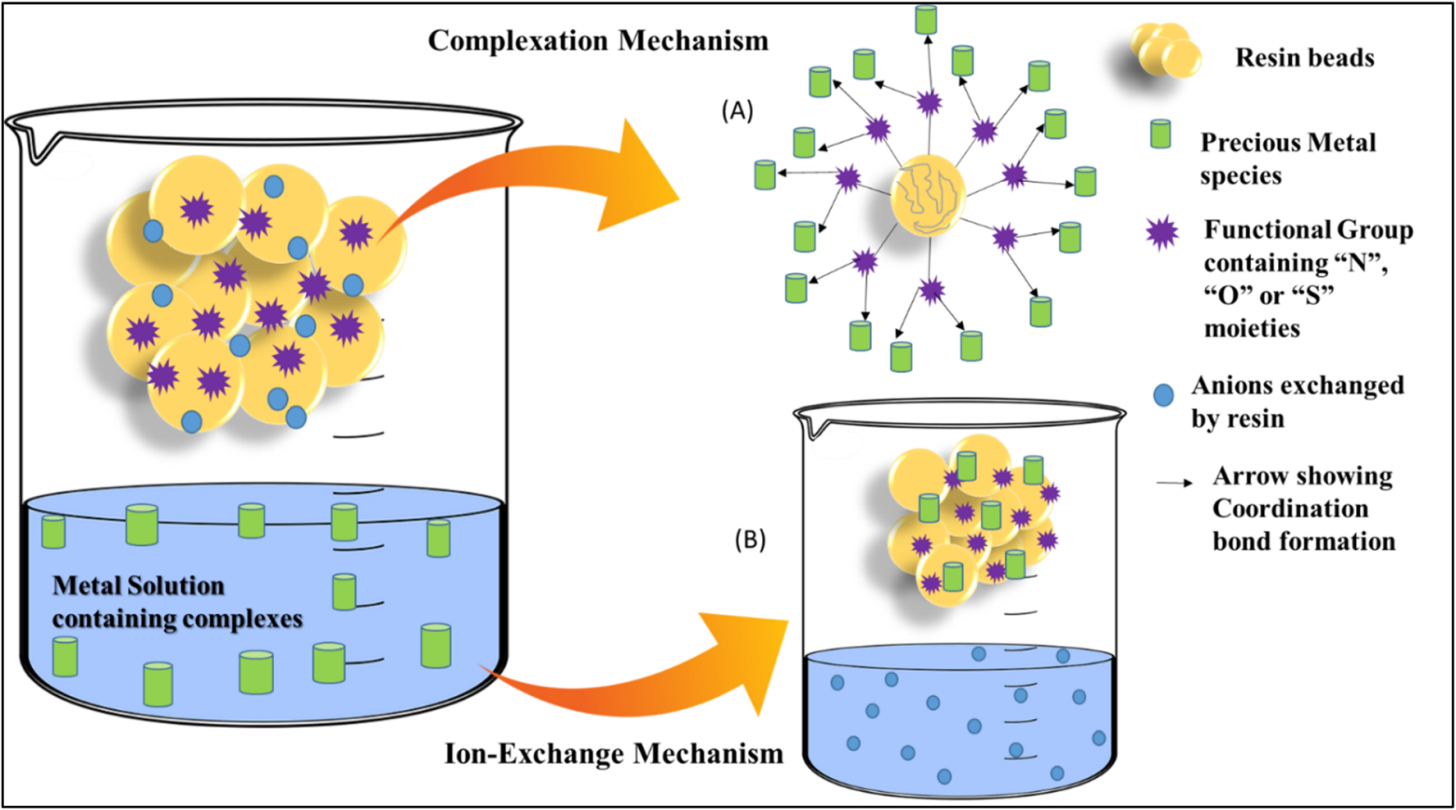
An Illustration of dual adsorption mechanisms of precious metal ions on functionalized resins. (A) Ion exchange *via* electrostatic attraction, (B) surface complexation through coordination with active donor sites.

Resins are functionalized polymers that are capable of exchanging ions in solution with ions attached to the resin, enabling selective adsorption and recovery. Depending on the functional groups such as thiols, amines, phosphines, or quaternary ammonium moieties, ion-exchange resins can target specific metal complexes through chelation, electrostatic interactions, or inner-sphere coordination as depicted in Fig. 2. Their reusability, selectivity and adaptability to varying pH and metal concentrations, make them a fundamental technique for precious metals recovery.

Gold cyanidation has historically dominated extraction owing to its operational maturity and economic efficiency, its intrinsic toxicity, environmental hazards, and limited efficacy on refractory ores have necessitated the exploration of alternative methodologies. Consequently, non-cyanide methods like thiosulfate leaching are gaining attention because they are safer and effective alternative due to their non-toxic reagents, rapid leaching, low equipment corrosivity, and tolerance to impurities such as carbon and copper. Dong *et al.* studied the adsorption behavior of gold onto Amberlite IRA-400, which showed the highest efficiency among the resins they studied. They showed adsorption occurring *via* ion exchange could be improved with higher resin dosage and higher pH (9–11) due to NH_3_ stabilization, where gold was adsorbed as [Au(S_2_O_3_)_2_]^3−^ replacing Cl^−^ on resin's functional group. Later they studied the synergistic role of Na_2_SO_3_ + NaCl in desorption from resin and converted [Au(S_2_O_3_)_2_]^3−^ to [Au(S_2_O_3_)(SO_3_)]^3−^. Recently they have shown Na_2_S_2_O_4_ can effectively reduce [Au(S_2_O_3_)(SO_3_)]^3−^ to elemental gold and have also addressed the effect of multiple circulations on gold leaching, adsorption, desorption, sulfur–oxygen impurity management, and process sustainability. Sulfate accumulation, produced due to the thiosulfate oxidation, highlighted the need to manage sulfur–oxygen impurity management and industrial sustainability. In another study thiosulfate solutions were explored to better understand how gold actually attaches to and releases from the resin. They noticed clear color changes and slight shifts in the spectra after adsorption, pointing to interactions between the metal and the ligand—essentially showing that complexes like [Au(S_2_O_3_)_2_]^3−^ were partly converted to [Au(S_2_O_3_)(SO_3_)]^3−^. XPS added another layer of detail by revealing how the oxidation state and coordination environment of the bound gold shifted during the process.^56–58^

Mahandra *et al.* and Liu *et al.* tested the selectivity and efficiency of gold recovery in acidic conditions by Thiocyanate pregnant leach solution (PLS) and thiourea-modified ZGA451 resin, respectively. Leaching provided a less toxic, single-step alternative to cyanidation without post oxidation neutralization. Selective Au(i) recovery from synthetic and real thiocyanate PLS was achieved up to 99% gold recovery at pH 2 with an effective Au–Cu separation. Whereas, thiourea@ZGA451resin showed excellent and selective Au(iii) adsorption *via* monolayer chemisorption involving electrostatic, chelation, and redox interactions. The process was spontaneous and endothermic, and the resin maintained high adsorption performance over up to five reuse cycles.^59,60^

Silver adsorption by the fabrication of tannin thiosemicarbazide formaldehyde (TTF) resin was studied by Sun. X., *et al.* which was prepared by Mannich reaction in order to incorporate nitrogen and sulphur groups into the framework for selective silver recovery. The increase in uptake efficiency of Ag ions, being a soft acid, was attributed to the synergistic role of inner sphere coordination, multi dentate chelation and acid-base controlled surface activation. The presence of atoms such as S, which act as Soft Lewis bases, nitrogen, which contributes *via* coordination through amine and imine groups to stabilize silver complexes, and oxygen atoms from hydroxyl groups present in tannin promoted adsorption efficiency through hydrogen bonding and other weak interactions. Kinetic studies revealed the rapid uptake of Ag ions with 84% removal efficiency by following multilayer adsorption mechanism.^31^

To reveal the impact of nitrogen and sulphur functional groups grafted on resins to adsorb silver ions, Liao *et al.* studied two thiourea-based resins, D840, TMMR and a thiosulfate resin with tertiary amino anion (D301) to observe their adsorption mechanism from photographic wastewater. Structural and chemical analysis of resin surface before and after adsorption revealed that adsorption mainly occurred by chelation and electrostatic interactions. In comparison with the other tested materials TMMR which was prepared by grafting thiourea on chloromethylated matrix exhibited the excellent performance achieving maximum uptake capacity of 602.1 mg g^−1^ with adsorbent dosage of 1.5 g L^−1^ at room temperature owing the dual binding mechanism as it offers Ag–N and Ag–S binding and further supported Nitrogen protonation under slightly acidic conditions (pH 2–5), which reinforced the electrostatic attractions toward Ag^+^. On contrary, D301 exhibited weak ion exchange mechanism rather than chelation whereas D840 demonstrated limited access to active sites.^61^ TUD@AXD7 designed by Mahmoud *et al.* exhibited adsorptive silver recovery followed by nanomaterial fabrication making a significant contribution to green chemistry, and material innovation with excellent stability and multiple reusability. The material with antioxidant and antifungal abilities exhibited a remarkable uptake capacity of 345.5 mg g^−1^ at pH 5, owing to the presence of thioamide and sulfonic groups that contribute electron sharing between Ag and donor atoms of nitrogen and sulphur. After adsorption, silver was desorbed as AgNO_3_ and then reused for the synthesis of stable AgNPs of regular morphology using trisodium citrate as the reducing agent underscored its cost effectiveness and operational resilience.^62^

Palladium recovery by Zhang *et al.* provided valuable insights into silica supported functional resins referred as SiAcyl and SiAaC developed through *in situ* polymerization and surface grafting strategies respectively. They demonstrated high loading capacity with good porosity and enhanced surface activity owing to ion exchange between Pd^2+^ and protons (H^+^) from the carboxylic acid groups present on resin matrix. SiAcyl (silica-based material functionalized with acyl groups) achieved a maximum adsorption capacity of 92.6 mg g^−1^ while SiAaC reached a capacity of 121.8 mg g^−1^ demonstrating outstanding adsorption and selectivity for Pd(ii) attributed to its enhanced accessibility of carboxyl and carbonyl functional groups, highly mesoporous structure with lower cross linking restriction.^63,64^ To address the challenge of palladium recovery from low-concentration acidic chloride solutions, two thioanisole modified Merrifield resins (Resin-*para*-1 and Resin-*meta*-1) were synthesized by grafting the resins with dual nitrogen and sulphur functionality. Protonated amine groups (–NH_3_^+^) on the resin *via* ion exchange, can replace chloride counterions whereas, thioether sulfur atoms (–S–CH_3_) coordinate directly with the Pd(ii) center through HSAB principle, forming stable Pd–S bonds that enhance both selectivity and binding strength. Between the two variants, resin-*meta*-1 showed superior performance owing to favorable spatial orientation of Sulphur and –NH_2_ groups, which facilitates more effective Pd coordination by reducing steric hindrance.^65^ Similarly, Chen *et al.* synthesized a chitosan resin (CR) by reversed phase crosslinking method which revealed Pd(ii) uptake of 195.22 mg g^−1^ through chemisorption between Pd(ii) ions and protonated amine groups while maintaining over 99.8% efficiency and remarkable stability even after eight regeneration cycles.^66^

Matsomoto *et al.* recovered rhodium by 4-butylaniline-impregnated resins (BuIRs) that demonstrated strong affinity toward the [RhCl_6_]^3−^ complexes, enabling efficient recovery of Rh(iii) even from low-concentration acidic solutions where equilibrium shift among Rh(iii) chloro-complex species in hydrochloric acid proceeded slowly however, thermal treatment accelerated the equilibration process. The adsorption mechanism was attributed primarily to the strong electrostatic interactions and formation of an ion-pair between the positively charged 4-butylanilinium ions and the negatively charged rhodium chloro-complex species ([RhCl_6_]^3−^) in the acidic medium. The presence of the hydrophobic butyl chain facilitates the affinity of the resin for chloro-complex anions by creating a nonpolar microenvironment, increasing diffusion and stabilization of the adsorbed species within the resin pores. Moreover, π–π and weak hydrogen-bonding interactions in the aromatic amine structure of 4-butylaniline contributed further in the stabilization of the adsorption complex. The desorption using methanol in Soxhlet extraction not only disrupts the ion-pair interactions but also regenerates the functional sites for reuse, indicating the potential recyclability of the system.^67^ Similarly, Puromet MTS9600 (a commercially available resin) was evaluated for the separation of platinum group metals (PGMs). Despite successful uptake, the desorption of Rh(iii) was highly inefficient, recovering less than 4% of Rh(iii), indicating the strong interactions of the Rh complex with resin matrix owing to their stability and slow ligand exchange rates. Thus, the conventional ion exchange mechanisms that rely only on outer sphere electrostatic interactions are insufficient for rhodium recovery from mixed PGM systems.^68^ Rhodium recovery by quaternary-ammonium impregnated resins such as nitrolite failed in real leachate, underscoring the motivation for exploring impregnated resins.^69^ However, radiografted chelating adsorbents that contain nitrogen and amide-rich ligands reveal significant promise in Rh(iii) recovery with an adsorption capacity of almost (>74 mg g^−1^) due to their ability to form stable interactions with Rh complexes.^70^

### 4.6. Biosorbents

Biosorption is a more recent developed subset of adsorption which is carried out by the use of biomaterials and micro-organisms as bio sorbent and the environmental conditions required for the growth and activity of those microorganisms. They are sensitive to pH, temperature, acidity, moisture, initial concentration of solute and ionic potency. Biomasses used for biosorption are categorized into dead and alive groups. Dead biomasses are more successful due to their long-term usability, simpler mechanisms, and no dependency on environmental conditions. Biosorbents like algae,^71^ yeasts, fungi,^29^ bacterial cells,^72^ living plant fibers^73^ and crab shells^54^ are most commonly used.

Recovery of gold by biosorption is governed by redox reactions and surface complexations involving biologically active functional groups oxygen or nitrogen containing groups on the biomass. This simple yet elegant mechanism allows biological systems to act simultaneously as adsorbents, reducing agents, and stabilizers, converting ionic gold into stable nanoparticles under mild, eco-friendly conditions. Nascimento *et al.* studied fungal biomass (*Trichoderma harzianum*) whose cell wall macromolecules are rich in electron donating groups such as –OH, –COOH, and –NH_2_. First, Au(iii) ions attach electrostatically to the negatively charged sites on the biomass. Then, through the natural reducing ability of these surface groups, especially the hydroxyls and aldehydes in polysaccharides, the ions are gradually reduced from Au(iii) → Au(i) → Au^0^. The newly formed Au^0^ atoms start to cluster into tiny gold nuclei, which grow into spherical nanoparticles stabilized by the surrounding biomolecules.^29^ Pan *et al.* investigated acid-treated woody biosorbent, where sulfuric acid activation plays a key role by introducing new surface groups like sulfonic and carbonyl sites, on hydroxyl (–OH) groups of lignin and cellulose in the woody biomass thus increasing porosity. These changes make it easier for the surface to adsorb and then reduce Au(iii) ions. The reaction usually starts with gold complexes binding to oxygen-rich sites, forming a short-lived Au–O–C intermediate, which then undergoes internal electron transfer. This leads to the direct deposition of metallic gold on the surface. It is a coupled adsorption–reduction process that happens without the need for external reducing agents.^74^ Similarly, in the tannin-based biosorbent prepared from *Pinus patula* bark, the polyphenolic structures serve as both the “hooks” and “fuel” for gold reduction. The abundant hydroxyl groups and aromatic rings provide strong binding sites and also donate electrons through their redox-active phenolic systems. As a result, adsorbed Au(iii) is reduced to Au^0^ while the phenolic groups are oxidized to quinones, a self-regenerating redox cycle that leads to nanoparticle formation across the polymer surface.^73^ The microalga *Galdieria sulphuraria* offers yet another interesting mechanism. Its surface contains nitrogen-rich functional groups such as amines, amides, and imidazoles which readily coordinate with gold complexes in acidic, chloride-containing solutions. These nitrogen atoms form Au–N and Au–Cl bridges, stabilizing the adsorbed gold species. Then, through proton coupled electron transfer (PCET) reactions involving both nitrogen and sulfur groups (–SH, –S–S–), Au(iii) ions are gradually reduced to Au(i) and finally to metallic Au^0^ clusters. As the gold is reduced, the cell surface regenerates its binding sites, creating a continuous adsorption–reduction loop that sustains nanoparticle formation.^71^ Bio-adsorbents derived from *Citrus sinensis* peels were chemically modified by Eriki *et al.* to enhance surface reactivity and reducing capacity. The abundant hydroxyl and carboxyl functional groups on the modified peels provided active binding sites for Au^3+^ ions. Initially, electrostatic attraction and complexation between AuCl_4_^−^ and these donor groups form intermediates. Subsequently, the phenolic and aldehydic groups act as mild reducing agents, transferring electrons to Au^3+^ and converting it to Au^0^, which nucleates on the biomass surface with 98.4% reduction efficiency.^75^ Similarly, a biogenic route employing *Candida rugopelliculosa* fungal system, Au^3+^ exposure triggers an oxidative stress response that activates intracellular and extracellular biomolecules, particularly reducing polysaccharides and proteins. These biomolecules provide lone-pair electrons (from hydroxyl and amine functionalities) to reduce Au^3+^ → Au^0^*via* enzymatic and non-enzymatic pathways. Simultaneously, peptide backbones and glycoproteins stabilize the formation of Au nanoparticles through biocapping. The correlation between decreasing nutrient availability and enhanced AuNP yield supports that the reduction originates from a stress-induced metabolic shift that maximizes the production of redox-active compounds.^76^ Adhikari *et al.* studied *Haematococcus pluvialis* microalgae, particularly after sulfuric acid treatment, exhibited exceptional adsorption and *in situ* reduction of Au(III) ions even under highly acidic conditions. The treated biomass achieved nearly 100% Au(iii) removal, attributed to the exposure of functionalized carbonaceous sites and the formation of surface complexes that facilitated electron transfer to reduce Au^3+^ into elemental gold.^77^

For adsorption of silver ions, Zhang *et al.* introduced an innovative N,S co-doped hierarchical porous carbon (NSPC), synthesized from *Camellia Oleifera* shell. The synergistic effects of N and S heteroatoms, combined with the hierarchical pore architecture, promoted the formation of Ag_2_S and Ag^0^ during the adsorption process with an uptake efficiency of 480.5 mg g^−1^ and selectively too high that it removed nearly 100% of Ag^+^ from mixed-ion solutions. Its excellent reusability up to 9 cycles and resistance to anionic interference further demonstrate its promise for sustainable wastewater treatment.^54^ Similarly, a biosorbent derived from woody biomass, modified with concentrated sulfuric acid demonstrated exceptional efficiency as it introduces sulfonic (–SO_3_H) and hydroxyl (–OH) groups onto the biosorbent surface that serve as strong coordination sites for Ag^+^ and Ag(NH_3_)_2_^+^ complexes through electrostatic and chemical interactions. The Ag^+^ ions were first converted to Ag(NH_3_)_2_^+^, and then undergo redox interactions, allowing partial reduction of Ag^+^ to Ag^0^ on the surface which led to a remarkable uptake of 1285 mg g^−1^, from complex industrial waste streams.^78^ Islam *et al.* proposed a biochar derived from spent coffee grounds (SCG) exhibited exceptional Ag^+^ adsorption up to 49.0 mg g^−1^ with 99.9% removal due to its moderate surface area, abundant oxygenated groups (–OH, –COOH) originating from coffee's lignocellulosic matrix, and inherent organic compounds that facilitated Ag^+^ reduction to Ag^0^ nanoparticles thus offering an eco-friendly and circular-economy pathway for silver recovery.^79^ The acidified, delignated *Sargassum filipendula*, a brown alga, demonstrated dual-mode adsorption of Ag(i) through concurrent chemisorption and physisorption. The adsorption process involves a combination of ion exchange, chelation, and surface complexation, primarily governed by oxygenated functional groups within the alginate network. Spectroscopic analyses (XPS) indicated elemental Ag formation, suggesting that the network not only binds ions *via* electron-donating oxygen groups but also facilitates redox transformations through polarization and local electron density effects.^80^ Feng *et al.* introduced 2,5-dimercapto-1,3,4-thiadiazole-modified wheat bran (DMTD-WB) for highly selective recovery of Ag(i) from multi-metal wastewater due to abundant –SH functional groups onto the wheat bran surface, which serve as strong electron-donating sites, forming stable Ag–S coordination complexes. It demonstrated an uptake of 60.39 mg g^−1^ with an efficient recovery nearly 81% after five cycles.^81^

Rhodium recovery by the development of recombinant *P. pastoris* GS-R remarkably escalate adsorption and selectivity compared to unmodified biomass owing to the presence of Rh metal binding peptides which offered additional carboxyl and amino functional groups that promoted specific binding affinity. Unlike orthodox microbial adsorbents, GS-R demonstrates notably enhance adsorption capacity (142.11 mg g^−1^) and selectivity for Rh(iii), surpassing waste *P. pastoris* and other reported biosorbents such as *S. cerevisiae* and *Shewanella algae*.^3^ An innovative aspect of GS-R is the maintenance of adsorption performance under acidic conditions (pH 1.2), making it compatible with real electroplating wastewater. Importantly, its selectivity for Rh(iii) in polymetallic systems was noticeably improved (36.74% higher than waste *P. pastoris*), underlining the potential of engineered yeast in maneuvering complex effluents. However, the desorption efficiency remained substandard yielding <1.5% recovery, and up to 18–34% recovery even with strong acids. This limitation emphasize a critical barrier to empirical cyclic application, as recurring adsorption–desorption is necessary for industrial feasibility.^72^

### 4.7. Biopolymer chitosan

Chitosan is a derivative of chitin which is commonly found in fungi, insects, crab and shrimp shells. Chitosan is made traditionally by four steps from chitin, demineralization to remove calcium carbonate using acid, deproteinization to eradicate proteins using an alkali, decolorization to devastate pigments, and deacetylation remove acetyl groups from chitin using strong alkali like NaOH. These processes result in a product that is rich in amino and hydroxyl groups present in molecular structure of chitosan make it an excellent adsorbent for precious metals. However, it has weak acidic compatibility and offers low reusability. To address the challenges, it is often physically and chemically modified which can introduce new functional groups and cross-linking sites to enhance adsorption efficiency.^82–85^

Li *et al.* and Fan *et al.* recovered gold from thiosulphate leaching solution by chitosan such as chitosan modified with tetrabutylphosphonium ionic liquid CTS–TBUP modified chitosan(BIM-CS). CTS–TBUP uses tri-*n*-butylphosphine (TBUP) as a functional monomer to introduce phosphorus donor sites that bind strongly by ligand exchange with Au(i) ions wherein TBUP not only coordinates but also reduces Au(i) to elemental gold, confirming a dual adsorption reduction mechanism. While, ionic liquid modified chitosan system emphasizes anion exchange as the dominant mechanism because of the presence of butylimidazolium based ionic liquids that boost up the material's affinity for negatively charged gold–thiosulfate complexes, achieving up to 96.7% uptake efficiency, which was faster than Aliquat-336 impregnated CS,^28^ and efficient than pristine chitosan.^86,87^ Wu *et al.* studied a bifunctional carboxymethyl Chitosan–tannic acid–graphene Chitosan aerogel (CCTG) which exhibited remarkable selective adsorption and reduction properties simultaneously with an uptake efficiency of 1761.6 mg g^−1^ at pH 3. Electrostatic interactions and tannin facilitated reduction converted Au(iii) to metallic Au(0). The appearance of yellow gold particles on the aerogel surface converted it into layered block like structure was due to the development of electrostatic interactions formed between AuCl_4_^−^ and protonated amines and other, chelation mechanism followed by nitrogen and oxygen atoms in chitosan with Au.^88^

A highly mesoporous adsorbent (Chitosan–Diaminomaleonitrile–Amidoxime) CS-DAMN-AO designed by introducing DAMN and amidoxime groups to create an open and interconnected pore network which increased the surface area and accessibility of active sites for Au(iii) adsorption achieving thermal stability by crosslinking of amidoxime and imine moieties, demonstrated maximum uptake of 1720.90 mg g^−1^.^89^ DACNF-CS demonstrated an adsorption capacity of Au(iii), and it could reach approximately 65.5 mg g^−1^.^90^ Lin *et al.* developed Quaternary ammonium (R_4_N^+^) functionalized chitosan fibers (QECFs) which demonstrated splendid maximum adsorption capacities of 669.75 mg g^−1^ at pH = 9.5 within 15 min, outperforming reported adsorbents in both capacity and rate.^91^ Silver ions have also been recovered from wastewater by the use of chitosan-based adsorbents. Liu *et al.* fabricated a zeolite-based chitosan magnetic composite (zinc-modified chitosan functionalized with maleic anhydride and tetraethylenepentamine) ZMC-MAH-TEPA through a green route, showing outstanding performance for silver attraction *via* electrostatic interactions and hydrogen bonding with –NH_2_ and –NH groups, with the advantage of magnetic stability. The recovery and reuse of the material was successful with high stability.^92^

A sustainable adsorbent (poly(acrylic acid)-grafted carboxymethyl-β-cyclodextrin-aminosilane composite) PAA-*g*-CMCDAS was derived from dialdehyde starch, polyacrylic acid, and carboxymethyl chitosan, demonstrating remarkable uptake of 404.77 mg g^−1^ for silver ions with more than 91% recovery efficiency after five desorption cycles.^93^ Another advancement was the synthesis of triazole-thiol functionalized chitosan (CS-AHMT) by Zhu *et al.* which exhibited the removal efficiency up to 241.4 mg g^−1^ at pH 5, with DFT-supported results revealing that electrostatic and coordination were the major driving forces behind silver recovery.^94^ Thermosensitive chitosan hydrogels made an advancement prepared through a sol–gel process using sodium β-glycerophosphate as a crosslinker. This material displayed high porosity and smooth structure that effectively adsorbed Ag(i) ions with 34% removal efficiency. The involvement of –NH_2_ and –OH groups was confirmed by characterization analyses which also revealed homogenous deposition of silver ions within the hydrogel network.

Similarly, Hamza *et al.* studied CFs-TU exhibits the lowest adsorption energy and strongest affinity for Ag(i) ions where advanced studies by DFT analysis revealed the coordination between Ag(i) and SH/C=S/C–N groups in addition to electrostatic interactions. Significantly, (cellulose fibers-thiourea composite) CFs-TU exhibit the strong reduction ability for converting Ag(i) to Ag(0).^95^ In continuation to develop thiourea modified resins, Lin *et al.* developed CFs-TU highlighting the limitations of traditional trial and error synthesis methods and developed CFs-TU as the most promising network due to their low adsorption energy with high selectivity and stability.^96^

Palladium ions have been extensively recovered by Chitosan-derived materials due to their biocompatibility, tunability, and structural flexibility. Kaolin/Chitosan hybrid nanofibers, built by using citric acid as a crosslinker, demonstrated an increase in adsorption performance from 31 to 64 mg g^−1^ after kaolin incorporation.^97^ Li *et al.* developed an acrolein-crosslinked chitosan hydrogel (A/CS) with a maximum uptake efficiency of 505.05 mg g^−1^, owing to the strong chelation between amino and hydroxyl groups of Chitosan with Pd(ii) ions in water.^98^ Furthermore, a chitosan resin (CR) synthesized *via* a reversed phase suspension using a two-step crosslinking approach showed an excellent adsorption capacity up to 195.22 mg g^−1^ with 99.8% removal efficiency even after eight cycles.^66^ An advancement was made by Arif *et al.* by the synthesis of core shell silica@poly(chitosan-*N*-isopropylacrylamide-methacrylic acid), S@P(CNM) microgel system for effective adsorption of Palladium, followed by *in situ* reduction of Pd ions. The degradation of different dyes such as MeB, MeO, RhB, and 4-NiP confirmed the multifunctional catalytic properties of palladium.^2^ Recently, Nagireddi *et al.* investigated the adsorption behavior of triethylenetetramine cross-linked chitosan (CH-TETA) for Pd(ii) recovery from water containing multiple complex ions like EDTA, NH_4_OH ions. Despite the presence of multiple ions, batch experiments showed the uptake efficiency of Pd(ii) ranging from 43.53 to 117.08 mg g^−1^, which corresponds to removal efficiency ranging from 39–87%. This study focused on the involvement of advanced regeneration techniques such as electrochemical, thermal, ultrasonic, *etc*, and appropriate pretreatments to remove interfering ions.^99^Table 2 summarizes the critical parameters governing chitosan-mediated adsorption of Ag, Au, Pd, and Rh from wastewater.

**Table 2:** Key parameters involved in the adsorption of precious metals (Ag, Au, Pd and Rh) by Chitosan from waste water

Precious metal	Chitosan/resin	Adsorption mechanism	Functional groups	Maximum adsorption capacity (mg g^−1^)	Initial concentration of metal in sample (mg L^−1^)	Temperature and pH	Contact time (minutes)	Isotherm and kinetic model	Selectivity for metal	Reusability	References
Gold	CTS-TBUP	Ligand exchange & reduction	PBu_3_, NH_2_	46.34	50	pH: 6–11 at 298 K	1440	PSO & Langmuir	Excellent	Upto 5	54
	ILs modified CS	Ion exchange	–NH_2_	5	10	pH: 6–7 at 298 K	20	PSO & Freundlich	Excellent	Upto 5	56
	CCTG	Electrostatic interaction, chelation, reduction	NH_2_, OH	1761.6	50	pH: 3 at 35 °C	80	PSO & Langmuir	Excellent	—	57
	CS-DAMN-AO	Chelation, reduction	–NH_2_, amideoxime	1720.90	800	pH: 5 at 318 K	60	PSO & Langmuir	Excellent	Upto 5	58
	DACNF-CS	Electrostatic interaction, chelation, reduction	Amino, hydroxyl groups	65	10	pH: 3 at 298 K	300	PSO & Langmuir	Excellent	—	59
	QECFs	Electrostatic and ion exchange	–NH_2_	669.75 ± 47.20	500	pH: 9.5 at 298 K	15	PSO & Freundlich	Excellent	Upto 5 cycles	60
	Thiourea@ZGA451	Electrostatic interactions	Thiourea, N and S atoms	∼100% removal efficiency	20–100 mg L^−1^	pH ≈ 3, room temperature	90–180 min to equilibrium	PFO, PSO	Excellent	Upto 5 cycles	60
	Nitrolite aliquat 336	Ion-exchange	Quaternary ammonium	73.49 mg g^−1^	10–1000 mg L^−1^	∼293 K	Up to 1440 min	PSO, Langmuir isotherm	Excellent	Upto 5 cycles	69
Silver	ZMC-MAH-TEPA	Electrostatic, hydrogen bonding	–NH_2_, –NH	70.12	50	pH: 5, temp 303 K	60	PSO & Langmuir	Excellent	—	61
	PAA-*g*-CMCDAS	Coordination	COOH, OH, NH_2_	404.77	600	298 K	30	PSO & Langmuir	Excellent	Upto 5	62
	CS-AHMT	Electrostatic, coordination	-OH, –NH_2_, –SH	241.4	100	pH: 5 and 318 K	60–120	PNO & Sips	Excellent	Upto 4	63
	Chitosan hydrogels	Coordination, electrostatic interactions	-NH_2_, –OH	—	—	298 K	1440	Sips	Excellent	—	64
	CH-TU	Coordination	Thiourea, –NH_2_, C=S	229.23	9.7–517	pH: 6 at 294 K	20	PFO & Sips	Excellent	Upto 20	65
	CFs-TU	Coordination, electrostatic interaction	–SH, C=S, C–N, –NH_2_	273.7	30	pH: 6 at 298 K	180	PSO & Langmuir	Excellent	Upto 5	66
	Chelating resins (D301, D840, TMMR)	Chelation and ion-exchange interactions	N, S donor atoms	∼133 mg g^−1^	∼200 mg L^−1^	∼298 K	Equilibrium within hours	PSO, Langmuir	Excellent	Upto 5	61
Palladium	CS/Kaolin-3	Coordination	NH_2_, COOH, OH	64	20	pH: 4 at 35 °C	60–120	PSO	Excellent	Upto 6	67
	A/CS	Chelation, electrostatic	NH_2_, –OH	505.05	110	pH: 4, temp 323 K	120	PSO & Langmuir	—	—	68
	CH-TETA	Coordination, chelation	NH_2_, NH	120	50–300	pH: 2 at 333 K	300	PSO & Langmuir	Excellent	—	69
	CH-ME	Coordination, electrostatic	NH_2_,NH	28	50	pH: 2 at 298 K	720	Freundlich & PSO	Good	—	70
Rhodium	Chitosan resin	Electrostatic attraction, chemisorption	–NH_2_, –OH	195.2 mg g^−1^	50–200 mg L^−1^	pH: 1–3, ∼25 °C	∼30 min for rapid adsorption	PSO, Langmuir	Excellent	Chitosan resin	66
	Nitrolite aliquat 336	Ion-exchange interaction	Quaternary ammonium groups	47.63–51.27 mg g^−1^	10–1000 mg L^−1^	∼293 K	Up to 1440 min	PSO, Langmuir isotherm	Moderate	Nitrolite Aliquat 336	69

### 4.8. Functionalized nanomaterials

Nanomaterials owing to their large surface area to volume ratio enable high adsorption capacities and faster kinetics. The reactive metallic sites or presence of functional groups such as –COOH, –OH, –NH_2_, allow metals ions to bind *via* surface complexation, ion exchange, electrostatic attraction or redox reactions. Nanoparticles can be engineered by polymer coatings, magnetic cores, or bio templates to improve selectivity and recovery from solution.^100^ Ganzoury *et al.* recovered gold by the use of Pristine MWCNTs, amide functionalized and carboxyl functionalized MWCNTs and concluded that Pristine MWCNTs exhibit higher affinity for Au from acidic AuCl_4_^−^ solutions mimicking acidic e-waste leachate.^101^ Zhang *et al.* recovered Ag, Au and Pd ions from wastewater under acidic conditions by the use of graphdiyne and its oxide derivative which belong to the class of two dimensional carbon based materials.^102^ Similarly, Chen *et al.* recovered gold by MoS_2_ nano-flowers from thio-sulphate solution.^103^ Recent studies highlight the importance of green nanomaterials, such as biomass-derived carbon and those synthesized from plant extracts, as alternatives to costly nanomaterials that pose toxicity and scalability challenges.

### 4.9. Metal–organic frameworks (MOFs)

**Fig. 3:**
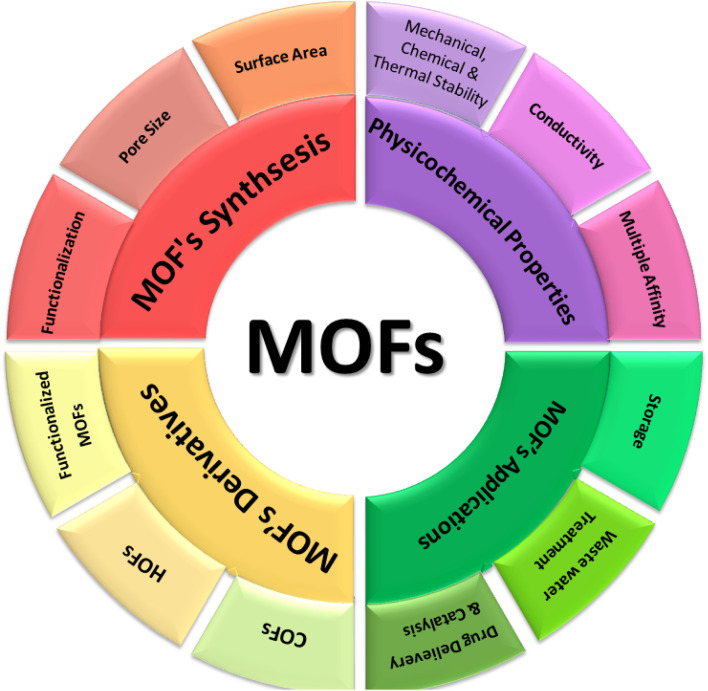
MOF's synthesis, properties, and major applications.

MOFs are hybrid three-dimensional materials with micropores having diameter less than 2 nm. Metal nodes typically Zr, Fe, Al, Ln create centers that link with organic ligands having carboxylate, hydroxyl, azolate, thiol or phosphonate functionalities to from a spongy crystalline framework of various geometries and features having rapid kinetics, high selectivity, and enhanced surface area. To complement the discussion, a schematic representation of the MOF's formation, properties, and functional scope is shown in Fig. 3. MOFs can be modified with soft donor atoms (S, O, N) and designed with size exclusion features because larger cavities, wider channels, and macroporous framework facilitate efficient diffusion and adsorption uptake. Strategies such as employing longer ligands, larger metal clusters, or introducing defects further contribute to pore size expansion.^104^

**Fig. 4:**
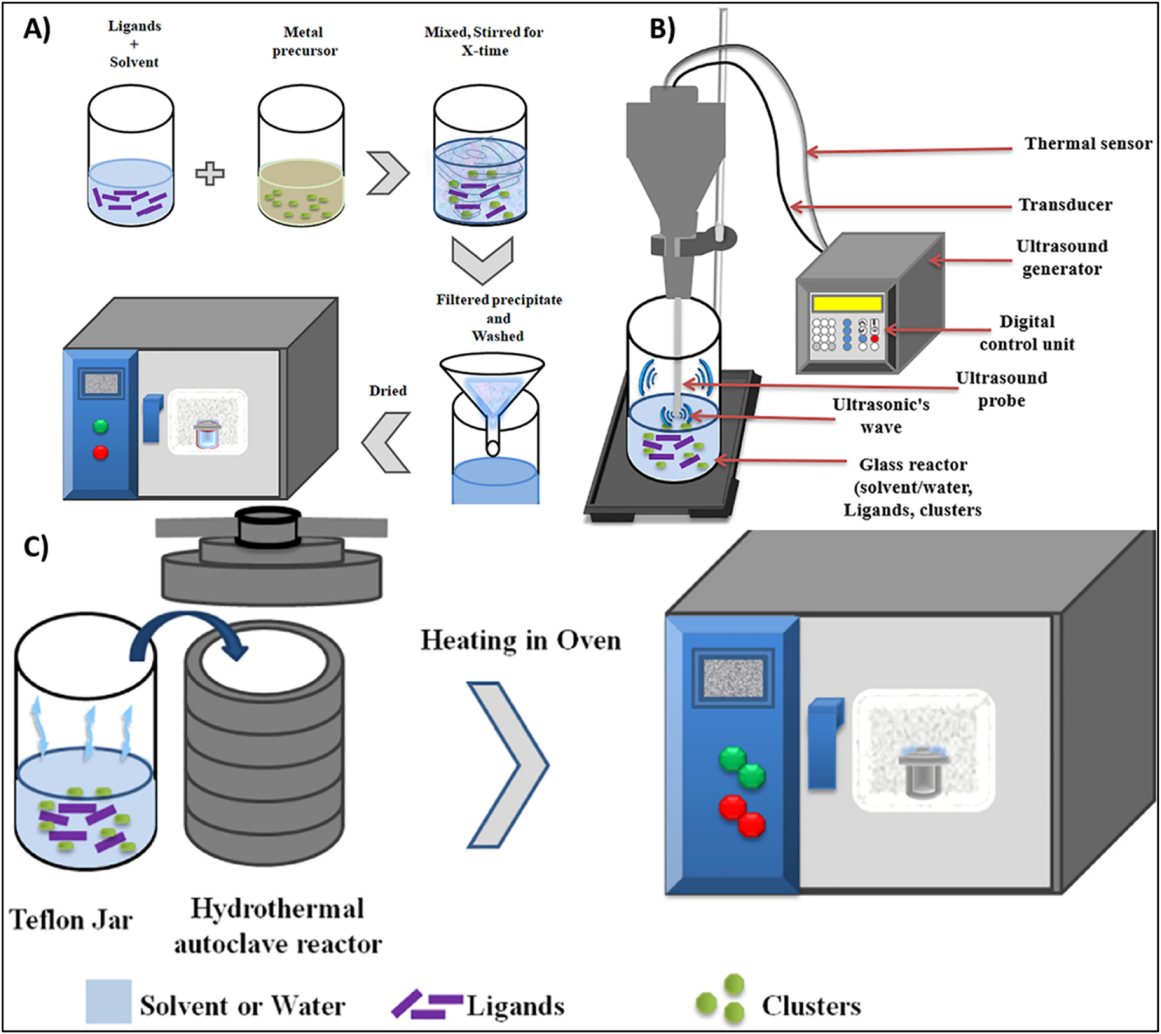
Common methods for the synthesis of MOFs (A) precipitation method, (B) ultrasound technique, (C) solvothermal method, reproduced from ref. 104 with permission from Elsevier Zeggai Z., Ait-Touchente Z., Bachari K., Elaissari A., *Chemical Physics Impact*, 2025, **10**, 100864, copyright 2025.

Across all MOF synthesis strategies (as shown in Fig. 4A–C) for precious metal recovery, solvothermal is most efficacious method due to the formation of highly crystalline and chemically stable MOF with high efficiency and selective adsorption. Microwave assisted MOF synthesis offers an energy efficient and faster alternative whereas mechanochemical and ultrasonic methods introduce average characteristics in MOF. Precipitation method, though cost effective and simple, generates subordinate crystalline frameworks with reduced selectivity.

For efficient and selective recovery of gold, Wang *et al.* developed UiO-67-MAA through an induced defect strategy, which significantly enhanced surface area, pore size, and density of active sites without gambling with thermal stability. The incorporation of methacrylic acid introduces sulfur-containing groups, facilitating the coordination of Au(iii) ions through S → Au interactions. XPS and EDS analyses indicated a chemisorption process in which Au(iii) is immobilized and subsequently reduced to metallic Au(0) *via* an electron transfer from the thiol functional groups.^34^ Similarly, Xiang *et al.* introduced the MOF-in-MOF approach by fabricating (metal–copper–1,4-benzenedicarboxylate–amino-functionalized)M-Cu-BDC-NH_2_,characterized by ultra-large pores that elevated resistance to diffusion and simultaneously enabled *in situ* reduction of Au(iii) to Au^0^, thereby sustaining high adsorption efficiency.^105^ Similarly zirconium-based metal–organic framework (MOF-808-DMSA), functionalized with dimercaptosuccinic Acid, features a porous MOF-in-MOF architecture that incorporates densely packed thiol groups derived from *meso*-2,3-dimercaptosuccinic acid. This design enhances the availability of coordination sites, which significantly increases the affinity for gold, facilitating the bonding of sulfur to gold and enabling the spontaneous reduction of Au(iii) to Au(i) and Au(0). DFT studies confirmed that these thiol and carboxyl groups act as the primary active centers, driving the strong chemical affinity and rapid adsorption kinetics, as its large interconnected channels allow gold ions to access active sites efficiently while maintaining open diffusion pathways for electron transfer.^4^

Chen's investigation on DONA-MOF underscored the importance of pore enlargement in improving selectivity and structural stability. The coexistence of multiple chelating sites and a Zr-based framework permits the formation of stable surface complexes through donor–acceptor interactions, while the sulfur and oxygen-rich chelates induce electron transfer to reduce Au(iii) to metallic Au(0).^106^ ZIF-8-MID, a thermally stable and non-toxic MOF, employs 1,3,4-thiadiazole linkers to generate electron-rich coordination centers that enable dual electrostatic and covalent binding with AuCl_4_^−^ species. The presence of the –SH groups promote partial reduction of Au(iii) to Au(i), concordant with a redox–chemisorption mechanism. Such collaborative action of electrostatic attraction, coordination bonding, and *in situ* redox transformation allows for efficient and selective Au(iii) capture even at low concentrations.^107^ UiO-66-AT (zirconium amidothiourea-benzenedicarboxylate MOF) depicted an improved uptake capacity (about 903.02 mg g^−1^ at 1000 mg L^−1^).^108^ Composite material such as carbon framework-supported UiO-66, metal–organic framework with dihydroxy-functionalized linkers synthesized using formic acid modulation CF-UiO-66-(OH)_2_-FA integrated MOF with cotton substrates are enriched in surface –OH groups, thus facilitating in controlled growth morphologies, tunable pore structures, and a high density of adsorption sites. It exemplifies how structural engineering promotes anchoring onto the MOF substrate that facilitates continuous monolayer chemisorption and redox cycling without material loss. This system achieved up to 99.9% gold removal efficiency, coupled with excellent processability, reusability, and facile recovery, thereby eliminating the necessity for ultracentrifugation.^109^ Similarly, Fe_1_Co_1_-MOF-74, was prepared by incorporating bimetallic sites and surface –OH functionalities, exhibited high adsorption capacity, rapid kinetics, and remarkable stability across a broad pH range. Its dual capability of reducing Au(iii) to metallic Au thus promoting gold nucleation and shedding has enabled recovery of high-purity gold with superior selectivity and reusability compared to pH-sensitive amine-based frameworks.^110^ Gold ions recovery by MOFs functionalized with amino groups, although frequently reported, demonstrate certain limitations due to their pH sensitivity, as low pH leads to the protonation of amine functionalities which in turn reduces adsorption efficiency.^30,111^

Silver ions can be effectively recovered by sulphur containing ligands *e.g.* UiO-66 when functionalized with thiol moieties exhibits more active sites for Ag due to Ag–S coordination.^112^ However other ligands containing nitrogen, oxygen or rhodanine (a Lewis base), derived groups can also form stable complexes while acting as soft donor atoms where silver act as soft Lewis acid. Rhodanine functionalization can transform an ordinary MOF into a dual donor site with flexible coordination framework. Ding *et al.* functionalized UiO-66(pristine) with rhodanine and developed (UiO-66-Rdp-m and UiO-66-Rdi-s) frameworks that exhibited exceptional adsorption capacities of 120 and 109 mg g^−1^, respectively, approximately sixfold higher than unmodified pristine UiO-66, owing to strong S–Ag chelation offered by the rhodanine (–C=S) functional group. The kinetics of post-modified MOF increased up to three-fold because of greater surface exposure, which enabled more sites for multidentate coordination.^113^ Similarly, MOF-RD, synthesized by grafting rhodanine-3-acetic acid with *p*-phthaldehyde and incorporating Zr clusters, was developed by Zhu *et al.* for selective silver recovery from industrial wastewater. The material exhibited amorphous texture due to non-uniform crystal growth caused by heterogeneous coordination between rhodanine linkers and metal centers which provided flexibility in structure, exposing more active sites and allowing rapid diffusion of silver ions through mesoporous channels. DFT calculations further imply partial charge transfer between Ag^+^ and the π-conjugated C=S/C=O network, resulting in localized electronic stabilization of the bound ions.^114^ Beyond UiO-type frameworks, MIL-type MOFs, also stand out for silver recovery. Ren *et al.* developed NH_2_-MIL-125 as an adsorbent for the removal of silver ions from simulated silver-plating wastewater, which proved highly selective and effective in the recovery of Ag in the presence of competing ions. The underlying adsorption is due to the incorporation of –NH_2_ functional groups into the MOF framework that enhances the electron density around Ti–O clusters, thereby strengthening the electrostatic attraction toward Ag^+^ and facilitating the Ag–N coordination. DFT calculations confirm that these amine sites provide high-energy coordination centers where Ag^+^ undergoes partial charge transfer, resulting in strong chemisorption. The implementation of fixed-bed column system demonstrated its strong competence for commercial applications.^115^ Mousavi *et al.* developed a magnetic MIL-101(Cr)-based composite (MIL-101(Cr)/Fe_3_O_4_@SiO_2_@2-ATP) and observed its sensitivity toward silver ions as it combines the porosity and adsorption capability of MIL-101(Cr) with the magnetic recoverability of Fe_3_O_4_ and the strong Ag^+^ affinity for thiol (−SH) and amine (–NH_2_) groups. The presence of 2-aminothiophenol (2-ATP) introduces both amino and thiol groups, which serve as strong donor sites for Ag(i) coordination through N–Ag and S–Ag bonds. These functional moieties facilitate localized charge transfer and complex formation at the MOF surface, leading to enhanced binding affinity and selectivity toward Ag(i) over other competing metal ions. The rough and heterogeneous surface morphology, observed through SEM, further supports the formation of multiple active sites for Ag(i) uptake, ensuring both high capacity and rapid kinetics.^116^

Palladium adsorption onto AHPP-MOF was studied by Tang *et al.* which acts as a potential colorimetric indicator for the qualitative estimation of Pd(ii) in water and it can be visually monitored by the color change of the adsorbent. Pristine AHPP-MOF was pale yellow in color before adsorption, and it turned brownish yellow after adsorption due to electrostatic interactions and chelation between PdCl_4_^2−^ species and the functional groups within the AHPP–MOF framework. The nitrogen and oxygen atoms present within the MOF structure not only contributed to binding but also donated electrons for the partial reduction of Pd(ii) to Pd(0). FTIR spectra showed a shift in the position of the C=N and –OH peaks, which arises from the reduction of metal ions, consistent with previous studies.^117^ Feng *et al.* developed a green approach for the synthesis of MOF-based composite beads having a cross-linked network that demonstrated effective palladium recovery from industrial water. B-MOF exhibited outstanding recyclability, maintaining its adsorption efficiency even after 12 regeneration cycles.^118^ Fe_3_O_4_@SiO_2_@-MIP-202 as a bio-based magnetic zirconium MOF, was studied by Piri *et al.* and employed as an efficient adsorbent for the recovery of Pd ions from wastewater without requiring sample pretreatments with toxic solvents. Characterization techniques confirmed its high porosity and thermal stability with active Zr sites and hydroxyl groups that enabled the maximum uptake of 194.5 g mg^−1^. The adsorption performance was tested on well water, groundwater, and municipal wastewater, which showed 95% recovery of Pd ions, highlighting its highly selective performance, making it a promising candidate for practical Pd(ii) recovery.^119^ Similarly, a stable, acid-resistant Si@-UiO-66-SO_3_H MOF was modified for the rapid extraction of Pd(ii) from acidic water, whereas unmodified UiO-66-SO_3_H degraded in acidic medium. The adsorption mechanism was attributed to the monodentate coordination between the sulfonic acid group and with Pd(ii) species, along with hydrogen bonding interactions.^120^ Xie *et al.* designed a covalent organic framework (COF) with an innovative single ion trap strategy. Azine-linked COF or ACOF-1 selectively recovered Pd due to the integration of hydrazine with carbonyl and pyridine functional groups in the framework, acting as a Lewis base, thus enabling efficient uptake of Pd even in a highly acidic environment. The framework demonstrated high exposure of active sites and structural stability due to antiparallel stacking that led to open ion trapping cavities, enhanced stability, and stronger Pd(ii) binding *via* N and O donor sites. The incorporation of redox-active linkers or bidentate donor moieties, can further improve the capture efficiency and recyclability.^121^

Rhodium recovery from wastewater is more challenging than any other precious metal, owing to its low occurrence and the inertness of its complexes. However, it is indispensable in industrial sectors such as catalysis, electronics, medicine, and petrochemicals, with its dominant application in three-way catalytic converters, where Rh(iii) enables efficient reduction of NO_*x*_. MOF, when functionalized with sulfur and nitrogen-rich ligands (*e.g.*, amines, amides, and dithiocarbamates), which upgrade binding affinity by HSAB principle, offers high selectivity for rhodium. Thiol-functionalized zirconium MOF (MOF-808(Zr)-Tz), was developed *via* grafting 2-mercapto-4-methyl-5-thiazolacetic acid (H_2_Tz), by Moghaddam *et al.* for the selective extraction of Rh(iii) from aqueous systems. The high surface area and porosity of the material provided swift uptake within just 1 min, followed by complete elution in 3 min. At neutral pH, the MOF surface is decorated with negatively charged –SH and thiazole groups that act as soft donors thus favoring the cationic Rh(iii) species whereas carboxylate donors offer additional binding stability. The demonstrated selectivity, rapid kinetics, and high recovery rates of MOF-808(Zr)-Tz, and as a reliable sorbent for Rh(iii) make it highly effective for trace-level monitoring and recovery.^122^Table 3 summarizes key factors influencing the adsorption of precious metals (Ag, Au, Pd, and Rh) from wastewater using MOFs.

**Table 3:** Key parameters involved in the adsorption of precious metals (Ag, Au, Pd and Rh) by MOFs from waste water

Precious metal	MOFs	Adsorption mechanism	Functional groups	Maximum adsorption capacity (mg g^−1^)	Initial conc. of metal in sample (mg L^−1^)	Temperature and pH	Contact time (minutes)	Isotherm and kinetic model	Selectivity for metal	Reusability	References
Gold	MOF-808-DMSA	Chelation, electrostatic interactions	–SH, COOH	2008.4	1100	pH: 1–6; 303–323 K	60	PSO, Langmuir	Excellent	3 cycles	11
	UiO-67-MAA	Chelation, electrostatic interactions, ion exchange	–SH, COOH	814	100	pH: 4.0; 298 K	10–250	PSO, Langmuir	Excellent	5 cycles	18
	M-Cu-BDC-NH_2_	Chelation, electrostatic interactions, reduction	–NH_2_, COOH	1184	100	pH: 3.0; 23.8 K	3	PSO, Langmuir	Excellent	5 cycles	19
	DONA-MOF	Chelation, electrostatic interactions	–OH, NH_2_, COOH	637.5	100	pH: 2.0–9.0; 303–323 K	120	PSO, Langmuir	Excellent	5 cycles	20
	ZIF-8-MTD	Chemisorption, electrostatic interactions	NH_2_, –SH	1604.53	0–200	pH: 2; 298 K	1440	PSO, Langmuir	Excellent	3 cycles	21
	UiO-66-AT	Chelation, electrostatic interactions, reduction	NH_2_, –SH, OH	903.02	1000	pH: 4.0; 298 K	3	PSO, Langmuir	Excellent	5 cycles	22
	CF-UiO-66-(OH)_2_-FA	Chelation, electrostatic interactions	–NH_2_, COOH	190.98	200	pH: 13; 298 K	Very small	PSO, Langmuir	Excellent	4 cycles	23
	Fe_1_Co_1_-MOF-74	Chelation, electrostatic interactions	-OH	3078	300	pH: 1.0–9.0; 23.4–24.9 K	3	PSO, Langmuir	Excellent	6 cycles	24
Silver	UiO-66(–SH)	Electrostatic, coordination	−NH_2_, –SH	358.6	200	pH: 3–6 at 298 K	10	PSO, Langmuir	Excellent	Upto 6 cycles	27
	UiO-66-Rdp-m	Electrostatic, coordination	C=O and C=S	120	300	pH: 5–7	—	PSO, Dubinin-ashtakhov	Excellent	Upto 10 cycles	49
	MOF-RD	Electrostatic, coordination	–NH_2_, C=N	707.2	1300	pH 5 at 318 K	240	PSO, Sips	Excellent	10 cycles	28
	NH2-MIL-125	Electrostatic, coordination	–SH	192.5	5–100	pH: 5.6 at 298 K	60	PSO, Langmuir	Excellent	—	29
	MIL-101(Cr)/Fe_3_O_4_@SiO_2_@2-ATP	Electrostatic, coordination	NH_2_, –OH	103	0.2	pH: 6.2	13	PSO, Langmuir	Excellent	—	30
Palladium	AHPP-MOF	Electrostatic, chelation	C=N, OH	283.5	100	pH: 4 at 318 K	1440	PSO, Langmuir	Excellent	Upto 5 cycles	31
	B-MOF	Coordination, electrostatic interactions	–NH_2_ groups	334.68	300	pH 3.0 at 298 K	1440	PSO, Langmuir	Excellent	Upto 12 cycles	32
	MIP-202	Electrostatic, chelation	Zr, –OH	194.5	1000	pH: 7.4 at 298 K	10	—	Excellent	Upto 5 cycles	33
	Si@-UiO-66-SO_3_H	Complexation, hydrogen bonding	SO_3_H	248	1000	pH: 5 at 313 K	5	PSO, Langmuir	Excellent	—	34
	ACOF-1	Coordination, electrostatic interactions	Pyridine, carbonyl	412.9	25–500	—	1440	PSO, Langmuir	Excellent	—	35
Rhodium	MOF-808(Zr)-Tz)	Coordination, electrostatic interactions	Thiol, thiazole, COO^−^	—	Trace amount	pH: 7, at 298 K	1	—	Excellent	Upto 9 cycles	36

Comparison can be made based on analysed and cited data. A prevalent characteristic of MOF-based systems is the intentional integration of soft donor functions (*e.g.*, –SH, –NH_2_, and rhodanine groups), which provide great selectivity for precious metals *via* robust coordination contacts and, frequently, concurrent redox transformation. For example, thiol-functionalized frameworks like UiO-67-MAA and MOF-808-DMSA have better Au absorption because S → Au bonding and *in situ* reduction. On the other hand, rhodanine-modified UiO-66 derivatives greatly improve Ag adsorption because of strong S–Ag chelation. Amine-functionalized MIL-type MOFs also increase the capture of Ag and Pd by giving electrons and using electrostatic interactions. Even if they have certain features in common, there are also important distinctions in their structure and performance. For example, defect-engineered and MOF-in-MOF systems make it easier for pores to be accessed for diffusion to happen, while composite and magnetic MOFs make it easier to process and recycle. On the other hand, simpler or natural adsorbents, such as bentonite, have lower capabilities and stability. However, there are still some issues, for instance amine-based MOFs are sensitive to pH, they do not retain their shape well in harsh conditions, and it is hard to make them on a large scale. Hence, functional group engineering and pore structure optimization are important for performance.

## 5. Electrochemical methods for the enhancement of adsorptive recovery

### 5.1. Electrodeposition

Electrodeposition is extensively used for recovering precious metals, relying on the electrochemical reduction of dissolved metal ions at the cathode, which facilitates their deposition in a metallic form. The effectiveness of reducing metal ions such as Au(iii), Ag(i), or Pd(ii) depends strongly on the coordination environment, particularly in cyanide or chloride media. In such coordination media, the formation of stable metal ligand complexes causes the reduction potential to shift, and the kinetics of electron transfer become coupled with ligand dissociation steps. As a result, the deposition process is often controlled not only by applied potential but also by mass transport and interfacial phenomena.^9^ From an application standpoint, electrodeposition is attractive because it yields metals in their elemental form, often with high purity. This is one of the reasons it has long been used in hydrometallurgical operations.^123^

### 5.2. Electrocoagulation

Electrocoagulation is considered in conjunction with electrochemical recovery, although its role and underlying mechanism is fundamentally distinct. Rather than directly reducing metal ions, it operates through *in situ* generation of coagulant species *via* the anodic dissolution of sacrificial electrodes, typically composed of iron or aluminum. The hydroxide species formed during this process act as scavengers, capturing dissolved metals through adsorption and co-precipitation mechanisms. Consequently, electrocoagulation is particularly advantageous for the treatment of aqueous streams containing metal ions in complexed, colloidal, or non-reducible forms, where traditional electrochemical reduction may exhibit limited efficiency.^10^ There is, however, an important limitation to acknowledge. Unlike electrodeposition, electrocoagulation does not produce a recoverable metallic phase directly. The metals are instead incorporated into sludge, which then requires further processing if recovery is desired. For this reason, it is often better viewed as a pre-treatment or concentration step rather than a standalone recovery method.^124^

### 5.3. Electrodialysis

Electrodialysis offers a more controlled route for separating ionic species, particularly when dealing with charged metal complexes. By applying an electric field across ion-exchange membranes, it becomes possible to selectively transport species such as Au(CN)_2_^−^ or PdCl_4_^2−^ into concentrated streams. A key factor of this approach is its inherent selectivity. In principle, the separation can be tuned through membrane choice and operating conditions. In practice, however, issues such as membrane fouling, scaling, and energy consumption tend to limit long-term operation. Recent work has explored hybrid configurations, where electrodialysis is combined with downstream electrodeposition or adsorption. These integrated systems appear to be more promising, particularly for moderately dilute streams.^125^

### 5.4. Electrokinetic transport and electroosmosis

Electroosmosis is often overlooked in discussions pertaining to precious metals recovery, yet it plays a crucial role in enhancing transport processes. The application of an electric field across a charged interface induces fluid movement which facilitate the migration of dissolved species toward reactive zones. While electroosmosis alone is not likely to be the main recovery method, it can significantly enhance mass transport in porous or nanostructured systems, especially under conditions where diffusion is limited. This is particularly important in low-concentration solutions, where transport rather than reaction dictates the overall efficiency.^11^

## 6. Electrochemically assisted adsorption systems

**Fig. 5:**
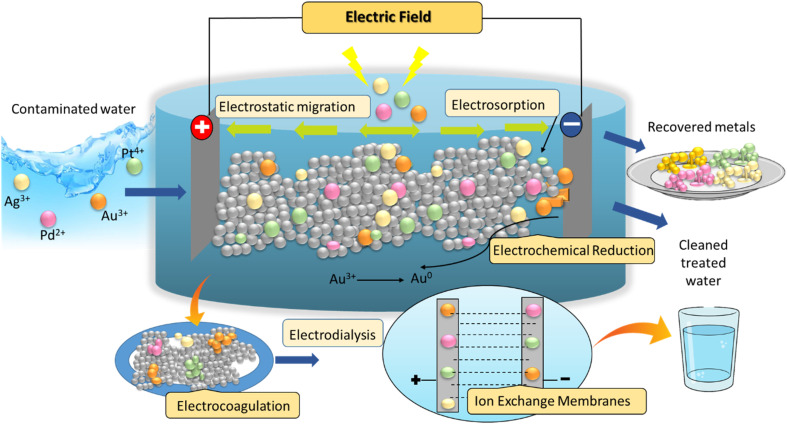
Schematic illustration of the electrochemically enhanced adsorptive extraction for recovering valuable metals from wastewater. By applying an electric field, this technique encourages the migration of ions, promotes electrosorption, and facilitates the electroreduction of metal ions to their elemental states on electrode surfaces, significantly improving recovery efficiency. The integration of additional methods such as electrocoagulation and electrodialysis enhances both separation efficiency and selectivity, ultimately leading to the production of treated water and the recovery of metallic products.

The integration of electrochemical and adsorption methods greatly improves the effectiveness of recovering precious metals, as shown in Fig. 5. The introduction of an external electric field prompts positively charged metal ions to migrate towards the cathode, influenced by electrostatic forces, thereby efficiently surpassing the diffusion constraints commonly linked with traditional adsorption methods. This movement increases the local concentration of metal ions adjacent to the adsorbent surface, facilitating electrosorption which is a field-enhanced adsorption phenomenon that enhances both the kinetics and capacity of absorption.

Subsequent to adsorption, electrochemical reduction transpires at the cathode, wherein metal ions acquire electrons and are transformed into their zero-valent metallic form, facilitating direct recovery through electrodeposition. The successive combination of electrosorption and electroreduction constitutes a synergistic mechanism that improves both selectivity and recovery efficiency. Simultaneously, supplementary electrochemical processes like electrocoagulation can promote floc formation and the trapping of residual metal species, whilst electrodialysis allows for selective ion transport across membranes. Collectively, these mechanisms demonstrate how electrochemical techniques can overcome the intrinsic limitations of standalone adsorption systems.

**Fig. 6:**
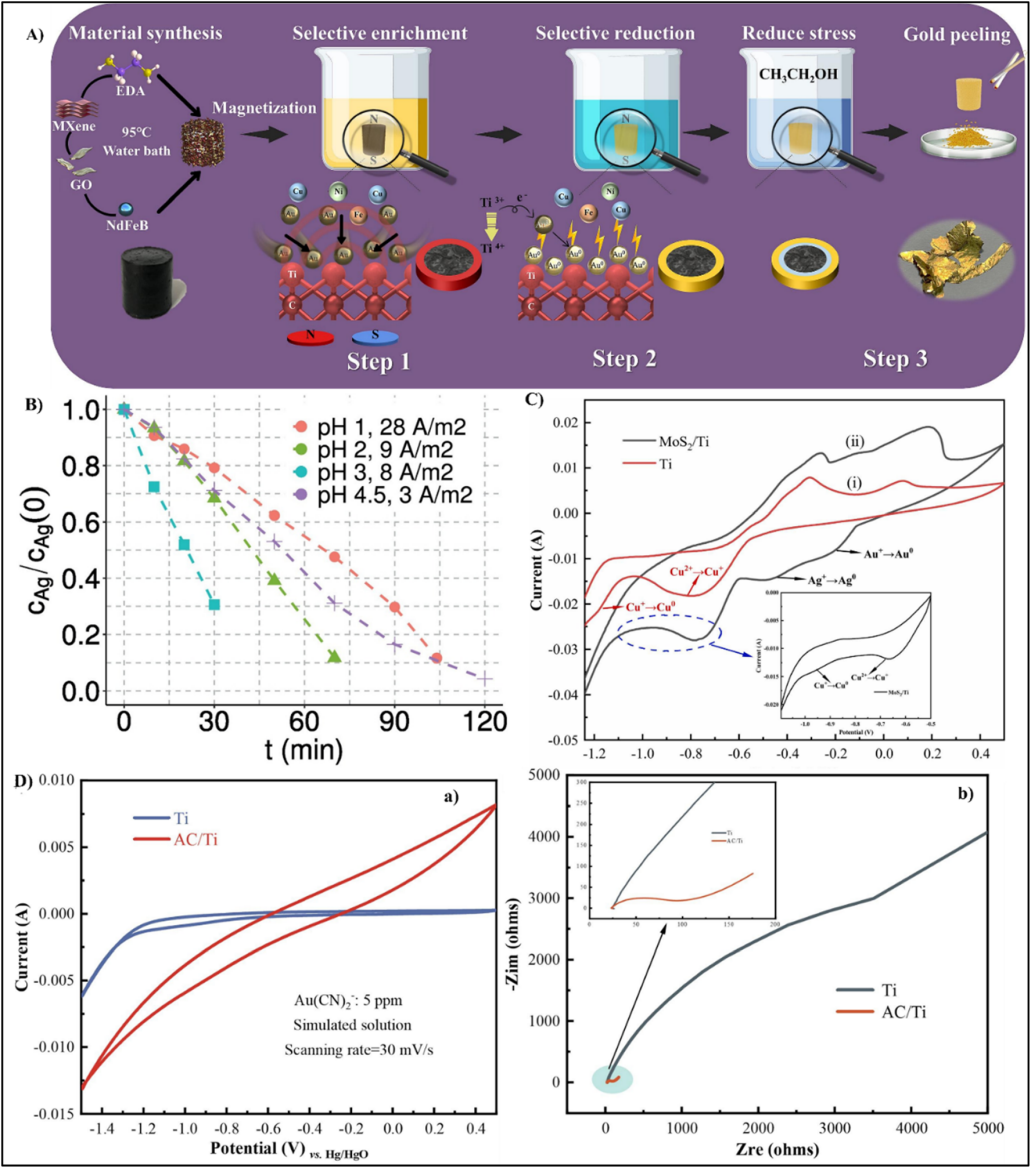
(A) Visual schematics of MMGH synthesis and gold recycling process, reproduced from ref. 12 with permission from Elsevier, Ren J., Zhu Z., Qiu Y., Yu F., Zhou T., Ma J., Zhao J., *Chemical Engineering Journal*, 2024, **479**, 147585, copyright 2024. (B) Silver removal by electrodialysis at different pH levels, reproduced from ref. 125 with permission from Elsevier, Zimmermann P., Wahl K., Tekinalp Ö., Solberg S. B. B., Deng L., Wilhelmsen Ø., Burheim O. S., *Desalination*, 2024, **572**, 117108, copyright 2024. (C) Cyclic voltammetry at scan rate (30 mV s^−1^) on MoS_2_/Ti and Ti, and (D) (a) cyclic voltammetry graph (b) EIS curves of activated carbon-coated titanium electrodes, reproduced from ref. 126 with permission from Elsevier, Wang D., Liang Y., Zeng Y., Liu C., Zhan C., Chen P., Song S., Jia F., *Journal of Hazardous Materials*, 2024, **465**, 133430, copyright 2024.

Ren *et al.* studied self-driven electrochemical recovery of gold by magnetically activated MXene/GO hydrogel (MMGH) that possesses magnetic, adsorptive, and reducing properties and demonstrated remarkable performance in highly acidic environments, bypassing the use of alkalis and other chemical treatments of acidic water. Actually, the surface of MMGH is positively charged in acidic medium, which could effectively capture AuCl_4_^−^ with an adsorption efficiency of 3321.3 mg g^−1^ at pH 2. After the selective adsorption of gold among competitive ions, the adsorbent offered physical stripping as shown in Fig. 6A, thus recovering gold without utilizing any toxic chemicals. EDS map of MMGH showed that the gold was adsorbed only at the surface, as shown in Fig. 6A, which is additionally beneficial for appropriate recovery.^12^

Wahl *et al.* studied the reducing behavior of MoS_2_/Ti cathode in low-concentration Au(S_2_O_3_)_2_^3−^ and Ag(S_2_O_3_)_2_^3−^ species in Cu–ammonia–thiosulfate leaching systems. The Ti electrode showed two cathodic peaks in corresponding to the sequential reduction of Cu^2+^ → Cu^+^ → Cu^0^. In contrast, the MoS_2_/Ti electrode exhibited four reduction peaks associated with Au^1+^ → Au^0^, Ag^1+^ → Ag^0^, Cu^2+^ → Cu^+^, → Cu^0^. In basic medium, as shown in Fig. 6B, the interference from H^+^ ions decreases, allowing more efficient migration and reduction of Ag^+^ ions while minimizing Cu^2+^ leakage.^125^Fig. 6D(a) and (b) confirmed the irreversible reduction behavior of Au(CN)_2_^−^ on the AC/Ti electrode, owing to its high capacitance and low charge transfer resistance. The synergistic interplay between adsorption and electroreduction significantly recovered 99.82% gold recovery within 24 h (Fig. 6B), outperforming absolute adsorption and electrodeposition by 34.47% and 81.66%, respectively, thus promoting ion diffusion toward the cathode and continuous Au(CN)_2_^−^ depletion near the surface.^126^

The same group developed an innovative adsorption and electrodeposition coupling (AEDC) mechanism by the use of activated carbon-coated titanium (AC/Ti) electrodes, which serves as a dual-function material simultaneously facilitating adsorption and electro-reduction of gold complexes to recover gold from industrial effluents. The higher cathodic peak currents on MoS_2_/Ti as shown in Fig. 6C, confirmed its high electron-donating ability and promoted metal reduction.^127^

Palladium recovery by electrochemical approach occurs through cathodic reduction of palladium ions (Pd^2+^) to metallic palladium (Pd^0^), where thermodynamics, PH, and kinetics play a crucial role. In an acidic medium, ample presence of H^+^ promotes charge neutralization and stabilizes the intermediate species contrarily, in basic medium, the competitive adsorption of OH^−^ on the cathode surface and the formation of hydroxo complexes such as [Pd(OH)_4_]^2−^ reduce the activity of ionic Pd. Electrode material and geometry further tune these effects. Using Graphite electrodes provides chemically inert, conductive surfaces that endorse uniform nucleation without adulteration, unlike stainless steel, which corrodes under anodic and acidic conditions, releasing Fe, Cr, and Ni ions. The electric field distribution between anodes and cathodes, if densely aligned in a sandwich-type configuration, promotes stronger Pd adhesion. By decreasing the thickness of the diffusion layer, hydrodynamic optimization accelerates ion transport to the cathode. Increased stirring at room temperature produces high recovery efficiencies, demonstrating that the process is limited by mass transfer rather than thermal activation. The process is highly competitive relative to precipitation, budget-friendly, and environmentally friendly, as it occurs without any emission of harmful gases.^128^

### 6.1. Electrochemical analytical and monitoring techniques

Techniques such as cyclic voltammetry and impedance spectroscopy are not merely diagnostic rather; they often guide the design of recovery systems. Cyclic voltammetry,^126^ for instance, provides insight into redox behaviour and allows identification of suitable operating potentials. In systems involving complexed gold or palladium species, it can reveal multi-step reduction pathways that are not immediately obvious. Similarly, electrochemical impedance spectroscopy (EIS) offers a window into interfacial processes, including charge transfer resistance and diffusion limitations. These parameters are particularly sensitive to electrode surface modifications, which are frequently employed to enhance selectivity.^126^

Pulse techniques such as differential pulse voltammetry (DPV) extend detection limits into the trace regime, making them valuable for monitoring recovery efficiency in dilute systems. Galvanostatic methods, on the other hand, are often preferred in practical operation due to their simplicity and ease of control.

### 6.2. Comparison of electrochemical and adsorptive approaches

Adsorption is more versatile when dealing with very low concentrations. It is relatively simple to implement and can achieve high removal efficiencies even in complex matrices. However, it typically requires a separate desorption step to recover the metal, which introduces additional complexity. Electrochemical methods, by contrast, offer a more direct route to recovery. The ability to convert dissolved ions into metallic deposits is a clear advantage, particularly from an economic standpoint. The trade-off lies in their sensitivity to concentration and operating conditions. In practice, the most effective systems tend to combine both approaches. Adsorption can be used to concentrate metals from dilute streams, after which electrochemical methods can be employed to recover them in metallic form. Such hybrid strategies are increasingly recognized as a practical way forward.

## 7. Analytical strategies for monitoring and understanding adsorptive performance

Morphological and structural characteristics of the synthesized materials are analyzed to evaluate their adsorption performance. An analytical characterization approach is employed, in which various instrumental techniques are integrated to examine the surface morphology, chemical functionalities, crystallinity, and elemental composition before and after metal adsorption. These analyses are essential for confirming the successful synthesis and modification of the materials, identifying the presence of active sites, and uncovering the structural changes that occur during interactions with metal ions. In many reports on adsorptive recovery of precious metals, material characterization is often limited to imaging techniques such as SEM and EDX. While these methods provide useful morphological and compositional information, they offer only a partial view of the factors governing adsorption behavior. In practice, adsorption performance is far more sensitive to parameters such as surface area, pore structure, and surface chemistry, which must be examined in a more integrated manner.

Electrochemically assisted adsorption represents a significant advancement in metals recovery strategies, bridging the gap between conventional adsorption and electrodeposition. Its effectiveness is strongly influenced by the interaction between the adsorbent material and the chemical environment. For example, the MMGH system reported by Ren *et al.* demonstrated exceptionally high adsorption even under strongly acidic conditions, avoiding the need for chemical neutralization.^12^ This is important to note because competing hydrogen ions typically limit many conventional adsorbents at low pH. Despite this advantage, the electrochemical contribution of the system is largely indirect, relying on self-driven reduction rather than sustained electrode activity. Although surface-localized deposition simplifies subsequent recovery, the lack of continuous electrochemical regeneration could restrict scalability in continuous or high-flow processes. Hybrid systems combine the benefits of adsorption with electroreduction. Due to synergism, continuous ion depletion at the electrode surface happens along with enhanced mass transport consequently increasing efficiency. Nevertheless, their performance remains highly sensitive to solution composition and competing ions. The dependency of the engineered electrode materials introduces practical challenges, including long-term stability, fouling, and fabrication complexity. The nature of surface functional groups is a critical factor in precious metals adsorption. Unlike common heavy metals, precious metal ions often exist as complex species, and their interaction with adsorbents is governed by specific coordination chemistry. Functional groups containing nitrogen, sulfur, and oxygen atoms are commonly introduced to enhance selectivity. Among these, sulfur-containing groups tend to show the strongest affinity for soft metal ions such as Au(iii) and Pd(ii).

A higher surface area generally provides more available sites for interaction; however, this does not necessarily translate directly into higher uptake unless those sites are chemically accessible and compatible with the target metal species. For many nanostructured adsorbents, particularly carbon-based and MXene-derived materials, surface areas can vary from tens to several hundred m^2^ g^−1^. Studies have shown that increases in surface area often improve adsorption capacity for Au(iii) and Pd(ii), but only when accompanied by appropriate functionalization.^129^ Thus, surface area should be interpreted alongside pore structure and surface chemistry rather than as an isolated parameter.

Porosity plays a more nuanced role than surface area alone. The size and connectivity of pores determine not only the accessibility of adsorption sites but also the rate at which metal ions can diffuse into the material. Micropores (<2 nm) contribute significantly to surface area but may restrict the diffusion of hydrated metal ions, particularly for larger complexes. Mesopores (2–50 nm), on the other hand, facilitate mass transport and are often more relevant for practical adsorption systems. Pore size distribution is typically derived from nitrogen adsorption–desorption isotherms using models such as BJH or DFT. A balanced micro–mesoporous structure is generally considered optimal, as it combines high capacity with efficient transport. Experimental observations consistently indicate that materials with hierarchical porosity exhibit improved performance, particularly under dynamic conditions.^130^

High surface area, suitable pore architecture, and tailored surface chemistry must work together to achieve both high adsorption capacity and selectivity. This interplay becomes particularly important when dealing with complex wastewater matrices, where competing ions and varying pH conditions significantly influence adsorption behavior. Consequently, a comprehensive analytical approach combining surface area, pore analysis, spectroscopic techniques, and electrochemical characterization is essential for rational material design.

## 8. Analytical and adsorption coupled studies

Zhao *et al.*'s research on the adsorption of gold mediated by fungal aerobic granular sludge was conducted at 25 °C with an agitation speed of 120 rpm, while maintaining a retention time of 2 h. The presence of AuNPs in solution was observed by using a UV-visible spectrometer with baseline normalization. The content of protein and glycan in extracellular polymers secreted by the fungi was measured by using the Lowry method and the phenol sulfuric acid method, respectively. Fungal cells were attained through centrifugal washing of both unmodified and modified fungal suspensions, a portion of which was dried and mixed with KBr for analysis of functional groups present on the surface using FTIR spectroscopy, where –OH, –NH_2_, –COOH, and other relevant groups were observed. After high-speed centrifugation and freeze drying, samples were treated for structural examination through SEM. The XRD results suggested that fungi secreted enzymes that were involved in the reduction of Au^3+^ to Au^0^.^76^

**Fig. 7:**
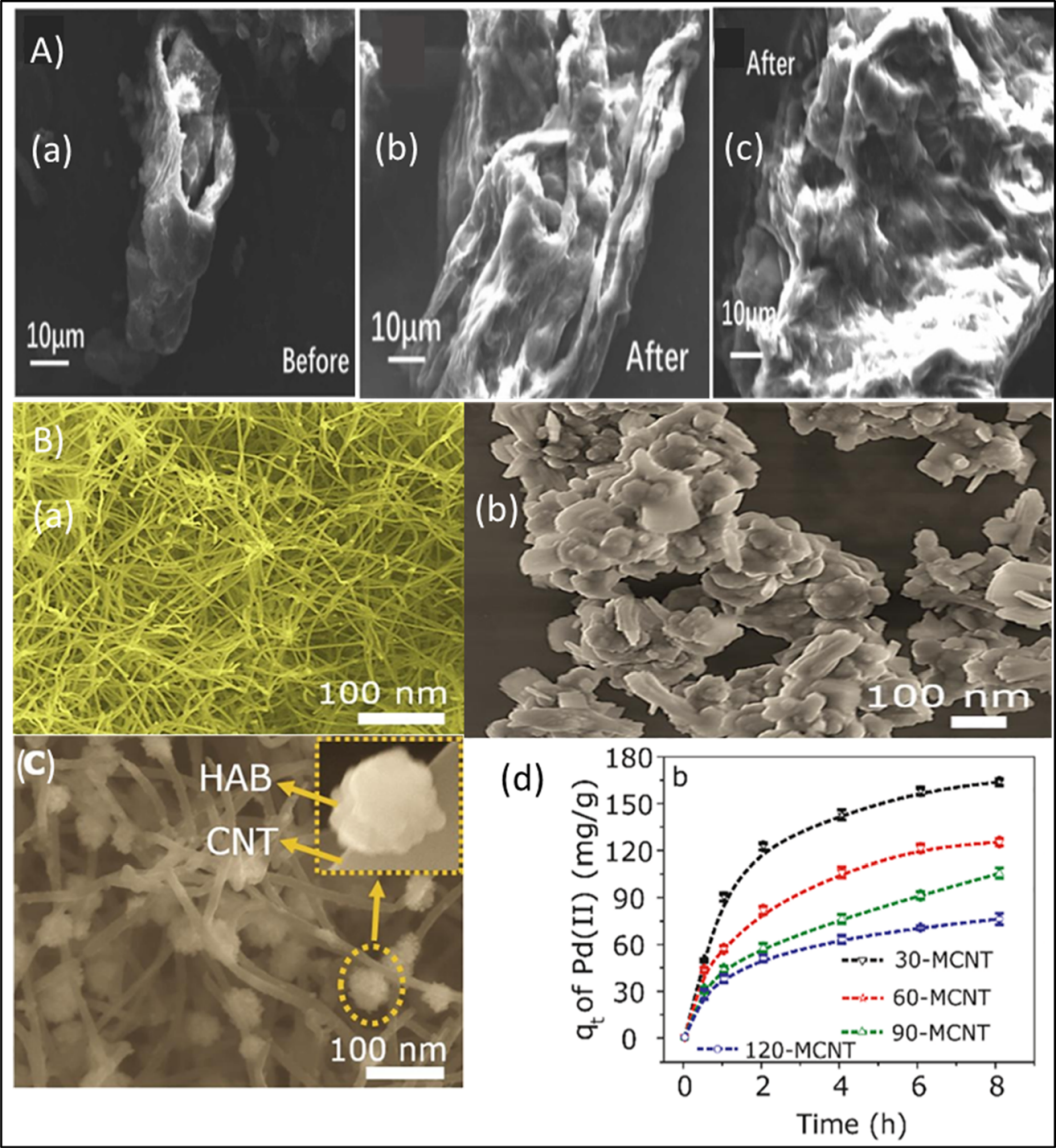
(A) SEM images of pomegranate peel adsorbent (a) before adsorption, (b) after adsorption for platinum, (c) after adsorption for Palladium, reproduced from ref. 131 with permission from Elsevier, Zhu Q., Zhu J., Huang K., *Journal of Environmental Chemical Engineering*, 2024, **12**, 111902, copyright 2024. (B) SEM images of (a) CNT sponge, (b) HAB-MOF, (c) 30-MCNT, (d) adsorption kinetics of Pd(ii) by the MCNTs, reproduced from ref. 132 with permission from Elsevier, Zuhra Z., Ali S., Ali S., Xu H., Wu R., Tang Y., *Chemical Engineering Journal*, 2022, **431**, 133367, copyright 2022.

Zhu *et al.* studied the uptake of gold, platinum, and palladium ions by using pomegranate peel (PP) as a biosorbent and evaluated it by using multiple analytical techniques. Functional groups like –OH, C–H, C=O, and C–O–C, which are characteristic of polyphenol-rich structures present in PP, were revealed by FTIR analysis. Changes in the spectra after adsorption indicated that –OH groups were oxidized while C–O bonds multiplied, revealing that phenolic groups were involved in the reduction of metal ions. SEM images showed a slightly smooth surface before adsorption; however, tiny gold, platinum, and palladium particles were observed on the surface after adsorption, as shown in the Fig. 7A(a)–(c). This successful metal deposition was further confirmed by EDS analysis, which showed the presence of O, Pd, Cl, and Pt on the surface. Protonated –OH ligands on the polyphenols electrostatically attracted negatively charged PtCl_6_^2−^, AuCl_4_^−^, and PdCl_4_^2−^ ions, which were subsequently reduced by π-electrons in the aromatic structure of polyphenols, were demonstrated by XPS measurements. Polarizing microscopy confirmed the formation of elemental gold, and XRD revealed the crystalline nature of other adsorbed metals, thus supporting a combined adsorption reduction mechanism. Eventually, quantitative analysis using ICP-AES revealed that the filtrate liquid contained minute quantities of metal ions, thus demonstrating the high selectivity and efficiency of PP in the adsorption of precious metals. These results depicted that pomegranate peel serves as an effective biosorbent that not only adsorbs metal ions *via* electrostatic interactions but also reduces them *in situ* to obtain elemental metals.^131^

Zuhra *et al.* studied the uptake of Au(iii) and Pd(ii) by 30-MCNT/HAB-MOF owing to its abundance of amino functional groups on the surface. FTIR analysis showed the shifts in peaks of –NH_2_, –NH, and –C–N after adsorption, confirming their involvement in binding with the metal ions. Morphological changes from a uniform distribution to aggregated particle structures after adsorption were examined by SEM, while EDX and ICP-OES measurements demonstrated selective uptake of Au(iii) and Pd(ii) efficiently. Kinetic studies showed metal uptake within 30 minutes, signifying effective covering of vacant active sites. The notable difference in adsorption capacities between HAB-MOF, CNT sponges, and 30-MCNT highlighted that the hybrid material provided more active sites, facilitated by the uniform distribution of MOF on the CNT sponge, which enhanced surface area and porosity for adsorption as shown in Fig. 7B(a)–(d). XPS analysis further confirmed complexation in sphere and electrostatic interactions assisted by protonation between amino groups and PdCl_4_^2−^, AuCl_4_^−^, PdCl_6_^4−^.^132^

Wang *et al.* conducted a comprehensive analysis of the synthesis and structural stability of UiO-67-MAA. They estimated the MMA loading capacity through X-ray diffraction analysis and investigated the incorporation of –SH groups and the coordination within the MOF using FTIR spectroscopy. Thermal stability was assessed *via* TGA up to 750 K. SEM images revealed a smooth surface with uniformly shaped octahedral crystals of UiO-67-MAA, while BET analysis indicated an increased surface area and pore volume, suggesting defect-supported porosity conducive to metal ion diffusion. The successful incorporation of thiol groups was confirmed through XPS and EDS, which detected the presence of carbon, oxygen, sulfur, and zirconium. Additionally, the high adsorption efficiency for Au(iii) was examined, revealing that factors such as pH, temperature, and concentration significantly influenced the adsorption process, as analyzed by ICP-OES. Zeta potential analysis revealed a positively charged surface promoting strong electrostatic attraction with AuCl_4_^−^ ions. Kinetic studies indicated a rapid adsorption, consistent with surface-to-pore diffusion behavior. EDS mapping showed that the absence of Au signals in the pristine sample confirmed the successful immobilization of gold ions onto the surface of the thiol-functionalized framework. XRD and SEM analysis of MOF after adsorption confirmed the preservation of the octahedral morphology and crystalline framework, indicating that the adsorption process did not alter the MOF structure.^133^

**Fig. 8:**
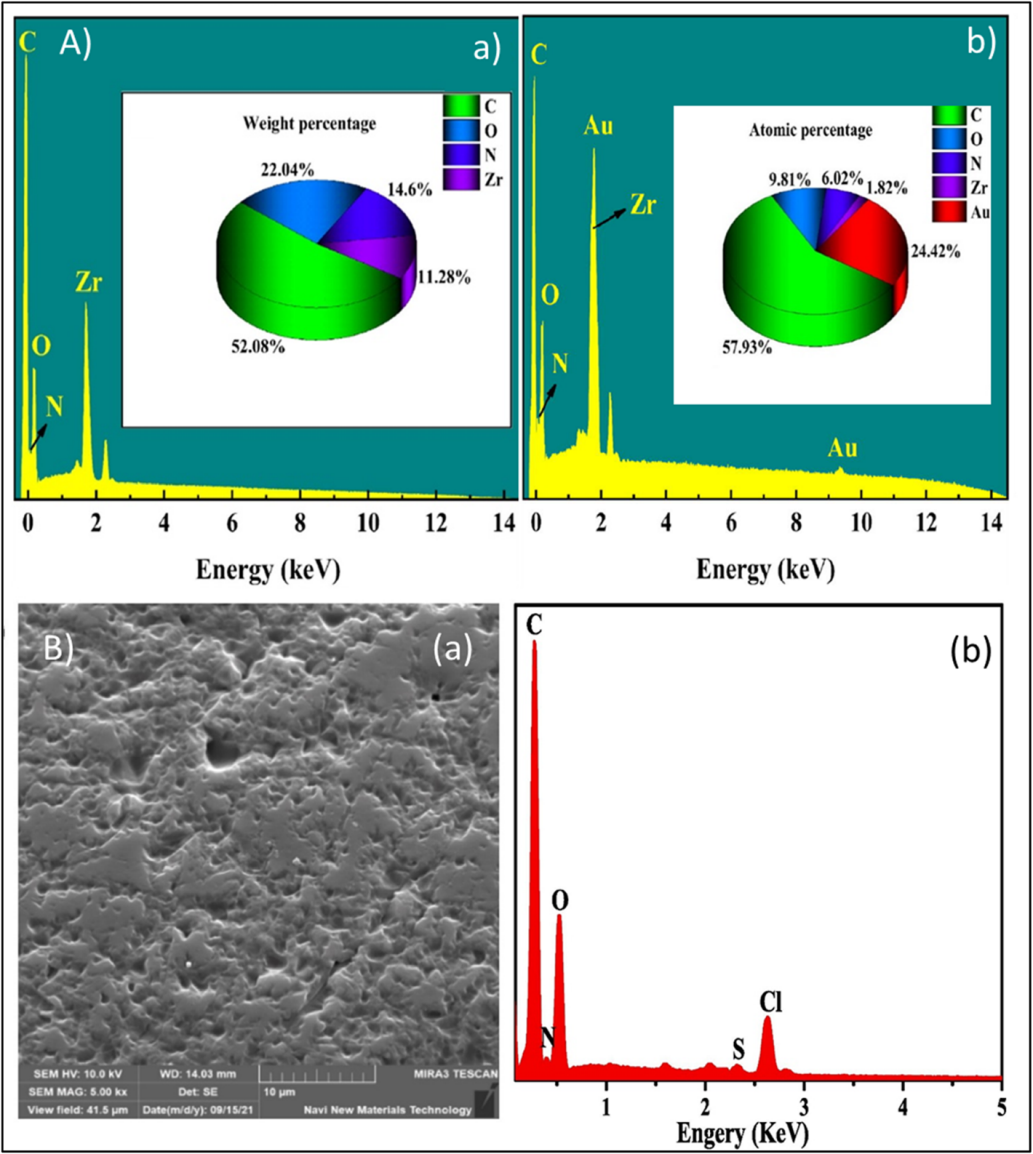
(A) EDS spectra and element weight percentages of DONA-MOF (a) before adsorption, (b) after adsorption, reproduced from ref. 134 with permission from Elsevier, Chen Y., Tang J., Wang S., Zhang L., *Journal of Molecular Liquids*, 2022, **349**, 118137, copyright 2022. (B) Barren resin (a) SEM image, (b) EDS spectrum, reproduced from ref. 56 with permission from Elsevier, Dong Z., Jiang T., Xu B., Li Q., Yang Y., *Separation and Purification Technology*, 2023, **323**, 124481, copyright 2023.

Correspondingly, Chen *et al.* analyzed DONA-MOF and observed its structural roughness and irregularity on the surface by SEM. XRD analysis revealed that the DONA-MOF possessed low crystallinity with noticeable structural defects, which increases the availability of active sites for adsorption. Pore size analysis by BET proved the efficient synthesis of MOF, whereas inner structure coordination was analyzed by FTIR. Water stability test demonstrated structural integrity across a broad pH range (2–10). The SEM images revealed a noticeable transformation in surface morphology, with the appearance of numerous white deposits identified as elemental gold through EDS analysis, as shown in Fig. 8A. The EDS spectrum revealed a distinct gold peak along with the characteristic signals of C, O, N, and Zr, thus confirming the effective adsorption of Au(iii) onto the surface. The appearance of prominent Au peaks in the XPS spectra after adsorption validated the incorporation of gold species within the framework.^134^

To gain deeper insight into the adsorption and desorption behavior of gold, Dong *et al.* investigated the thiosulfate solution (as described earlier). Characteristic color changes and spectral shifts after adsorption confirmed interactions between metal and ligand species, such as the transformation of [Au(S_2_O_3_)_2_]^3−^ to [Au(S_2_O_3_)(SO_3_)]^3−^. Furthermore, X-ray Photoelectron Spectroscopy (XPS) offered detailed information on the change in oxidation states and coordination environment of adsorbed metals. The appearance of Cl signals and the disappearance of Au peaks in the EDS spectra indicated that desorption occurred through ion exchange between Cl^−^ ions and the adsorbed gold complex. Morphological observations further revealed that the barren resin regained a smooth surface similar to the original form, confirming complete gold removal and preservation of the resin's structural integrity for reuse, as shown in Fig. 8B(a) and (b).^56^

Adsorbents can be comprehensively evaluated utilizing SEM, EDS, XRD, FTIR, XPS, BET, and ICP-AES to ascertain surface functionalization, active site accessibility, and structural integrity. Morphological, spectroscopic, and compositional analyses corroborate the adsorption-reduction mechanisms of these materials and their exceptional selectivity for valuable metals. Electrostatic interactions and *in situ* metal ion reduction transpire during adsorption on biosorbents (fungal muck, pomegranate peel) and hybrid metal–organic frameworks (MOFs), preserving the structural integrity of the material. These approaches provide the monitoring of desorption and recyclability for sustainable metal recovery.

## 9. Computational approaches for mechanistic insights

Computational approaches correspond to theoretical and computer-based modeling used to investigate adsorption mechanisms, predict performance, and optimize various factors without relying solely on experimental trials. Density functional theory (DFT), molecular dynamics (MD), Monte Carlo simulations, Machine Learning (ML), and Artificial Neural Networks (ANN) techniques not only help explain experimental observations but also enable the prediction and optimization of adsorbent materials for efficient metals recovery. In most cases, geometry optimization is carried out first to achieve the most stable configuration, followed by electronic density distribution analysis to estimate charge transfer, and after that, MD simulations are conducted to investigate thermal stability and compute adsorption energy values.

DFT serves as the cornerstone for studies on adsorption, offering valuable insights into charge distributions, the electronic structure, adsorption energies, and reaction barriers. Typically, adsorption energies and charge transfer calculations are performed following the optimization of geometry.^135^ Numerous computational modules within DFT are essential for optimizing molecular geometries and determining the adsorption energies, to provide practical guidance for adsorption mechanisms. In practice, plane-wave DFT codes such as Quantum ESPRESSO, VASP often employing GGA-PBE or similar functionals are common. A recent study by Heshami *et al.* focused on the interactions between gold and silver complexes with the G/GO surface, contributing to the understanding of their recovery processes.^136^

MD simulations provide insights into pore size distributions, hydration effects, adsorption isotherms, diffusion behavior and structural stability of adsorbate and adsorbent materials during adsorption processes. This computational technique helps in choosing suitable conditions to optimize adsorbent for specific applications. For instance, MD simulations of porous carbon have demonstrated that the adsorption by porous carbon materials is strongly influenced by solvent interactions and pore size distribution, which affect ion mobility and adsorption capacity.^137^

Monte Carlo (MC) methods statistically sample configurational space. Grand Canonical Monte Carlo (GCMC) is used for equilibrium adsorption isotherms especially in porous hosts.^138^ Kinetic Monte Carlo (kMC) is widely used for surface reaction kinetics and growth processes. MC uses a list of elementary reaction (adsorption, diffusion, reaction) rates to evolve the system randomly over times. For instance, Khnifira evaluated MC simulations to study the adsorption behavior of the heavy metals on graphite.^139^

Reactive force fields (ReaxFF) allow bond-breaking/forming within large-scale MD at moderate cost. ReaxFF has been parameterized for many metals and oxides.^140^ It can simulate complex reaction pathways on approximately 1000 atom cells in nanoseconds. Recent efforts focus on refining ReaxFF parameters with high-level DFT data to better capture defect kinetics and multistage transformations. In adsorption studies, ReaxFF can model, *e.g.* Yang *et al.* studied the interaction between zeolitic imidazole frameworks (ZIFs) and water.^141^

Machine learning (ML) significantly reduces experimental workload and enables rapid screening of new adsorbent materials. Supervised ML model such as regression is used to predict selectivity trends whereas, unsupervised method such as clustering identifies structure in large simulation datasets. Transfer learning and active learning loops may further improve efficiency. ML provides direct link to experiments, such as ML models for experimental work can predict removal efficiencies, adsorption capacities, and optimal conditions with high accuracy. For example, Li *et al.* developed an Artificial Neural Networks (ANN) model to predict the adsorption of Pd(ii) ions using functionalized biochar, achieving 95% prediction accuracy.^142^ Zhao *et al.* predicted adsorption capacity and its affecting parameters in the recovery of heavy metal.^143^ and Shu *et al.* predicted heavy metal adsorption by ML.^144^

**Fig. 9:**
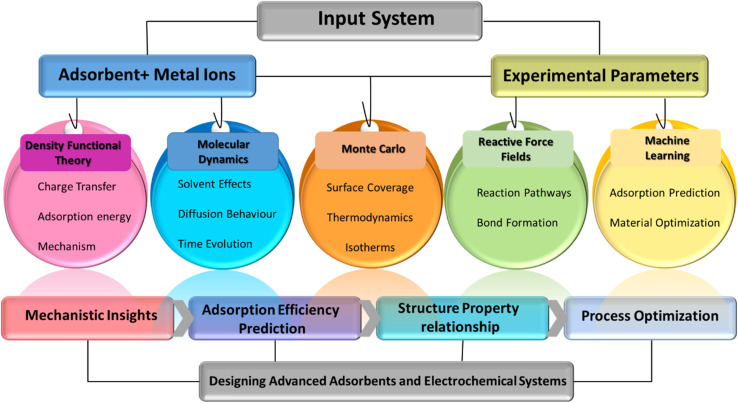
Computational approaches for mechanistic understanding of precious metals adsorption and recovery.

Computational modelling plays a crucial role in elucidating the fundamental mechanisms controlling adsorption and recovery of precious metals, as summarized in Fig. 9. Collectively, these computational approaches offer a multiscale framework that bridges theoretical predictions with experimental observations, facilitating the rational design of high-performance adsorbents and integrated electrochemical systems for efficient precious metal recovery.

## 10. Computational insights into adsorptive recovery

**Fig. 10:**
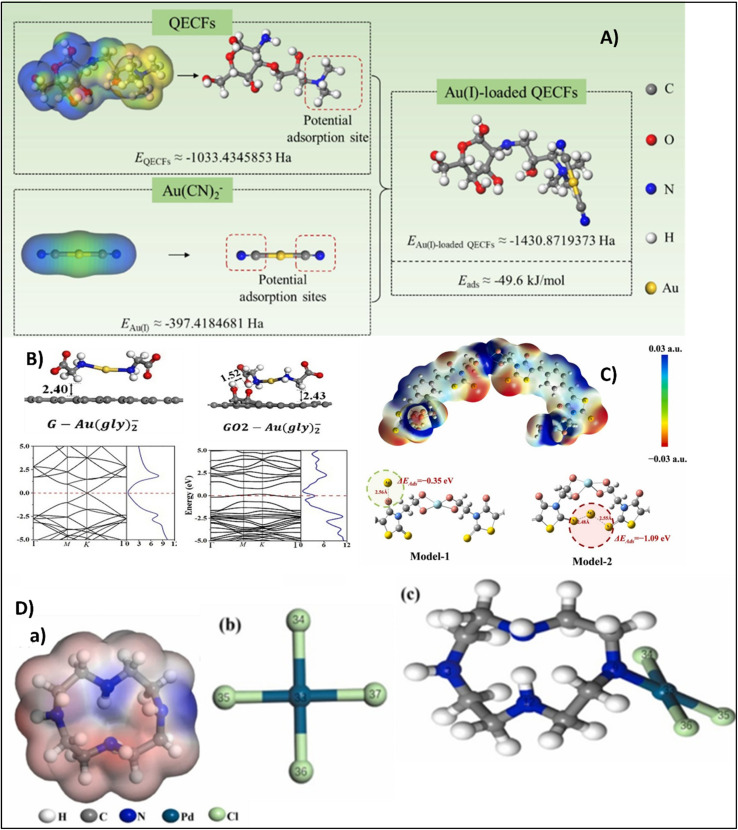
(A) Optimized configurations and MEP maps of Au(i), QECFs, and Au(i)-loaded QECFs, together with the estimated adsorption energies, reproduced from ref. 145 with permission from Elsevier, Lin X., Song M.-H., Lei L., Tran D. T., Shu Y., Lim C. R., Wu X., Mao J., Yun Y. S., *Separation and Purification Technology*, 2025, **362**, 131830, copyright 2025. (B) Band diagrams and TDOS for graphene and GO_2_ along with optimized arrangement of gold glycinate, reproduced from ref. 136 with permission from Elsevier, Heshami M., Tavangar Z., Taheri B., *Applied Surface Science*, 2023, **619**, 156676, copyright 2023. (C) Electrostatic potential distribution of MOF-RD and the two possible adsorption configurations between Ag(i) and MOF-RD, reproduced from ref. 146 with permission from Elsevier, H. U., Zhang H., Zhang C., Sun W., Han M., Wang R., Wei X., Li S., *Journal of Water Process Engineering*, 2024, **58**, 104779, copyright 2024. (D) ESP of Cyclen, structure of PdCl_4_^2−^, structure of Cyclen-PdCl_4_. Reproduced from ref. 147 with permission from Elsevier, Li Y., Tang S., Lei H., Zhong Y., Zhang Y. F., *Chemical Engineering Research and Design*, 2025, **214**, 166–176, copyright 2025.

Most of the studies on precious metal recovery are conducted through DFT calculations, which take into account all the ground state features as functions of electron charge density (*ρ*) and total energy (kcal mol^−1^). Lin *et al.* analyzed the mechanism of adsorption of quaternary ammonium functionalized chitosan fibers (QECFs) through DFT where van der Waals interactions were justified using by transition state (TS) method and adsorption energy or *E*_ads_ between Au(i) and QECFs were calculated theoretically revealing the process was exothermic and spontaneous. The Highest Occupied Molecular Orbital (HOMO) was also analyzed through DFT calculations indicated that the amino group in Chitosan has a strong tendency to donate electrons. In the Fig. 10A MEP map of QECFs, the blue and green zones depict negative electrostatic potential in the MEP map of Au(CN)_2_^−^, which are predominantly concentrated in the region containing the N atoms of the CN groups on Au(CN)_2_^−^. These results suggest that Au(i) is most likely to be trapped by QECFs through interactions between the R_4_N^+^ group on the QECFs and the N atom in the cyanide group on Au(CN)_2_^−^, on account of decreased DFT energy of this configuration, demonstrating the structure is thermodynamically more stable. This improved stability is caused by strong C–N bonding and even charge distribution, thus encouraging electrostatic adsorption of anionic pollutants rather than neutral or cationic species.^145^

Geometry optimization and theoretical analysis were executed on graphene to examine the adsorption of gold and silver glycinate by Heshami *et al.* using DFT by the DMol_3_, and the interactions between metal complexes and the G/GO surface were evaluated. To examine the electronic characteristics of these configurations, the corresponding band structure and total density of states (TDOS) diagrams are presented in Fig. 10B, where elucidated. Graphene's energy band profile shows convergence between the minimum conduction band (π*) and maximum valence band (π), which shows a zero-band gap, indicating that graphene is a semiconductor. However, hydroxyl and epoxy groups in the GO variant GO_1,_ being adjacent to each other, distort the π-electron system and withdraw or donate electrons and changing the material's electronic property. The formation of hydrogen bonds causes the adsorbed complex to move slightly away from the surface. An increased concentration of hydroxyl functionalities enhances the binding energy, as these groups facilitate charge transfer processes between the adsorbent surface and the adsorbate molecules.^136^

DFT calculations by Zhang *et al.* reveal the adsorption principle of Ag(i) on MOF-RD and galena surface. The results were recorded by Mulliken charge population, which manifested that N possesses a positive charge, and according to the electrostatic potential map as shown in Fig. 10C, it is located in an electron-rich zone. The negative charge on O functions as a clear index of their ability to donate electrons. S has a negative Mullikan charge, located in electron electron-rich region, thus reinforcing that coordination mainly occurred at the C=S sites. Optimized structure of Ag forms a stable Ag–S bond (2.34 Å) with an adsorption energy of −156.7 kJ mol^−1^, on galena surface, indicating a thermodynamically favorable interaction. Covalent character was confirmed by charge population and density analyses.^146^

Li *et al.* investigated the activity of Cyclen as an adsorbent and PdCl_4_^2−^ as an adsorbate. After associating with PdCl_4_^2−^, there was a contraction in bond lengths shown by Cyclen observed during DFT analysis, as shown in Fig. 10D. This adjustment may be ascribed to interactions between Cyclen and PdCl_4_^2−^. The nitrogen atom in the ligand (Cyclen) endures deprotonation, thus forming coordination bonds with metal ions. Computed adsorption energy confirmed chemisorption between the adsorbate and the active sites of the adsorbent.^147^ Similarly, Wang *et al.* calculated the binding energies of Pd(NO_3_)_2_ with S, N, and SH. All three binding energies were observed to be less than zero; however, the SH site turned out to be the most convincing adsorption site for Pd ions, with a notable adsorption energy of 2.63 eV.^148^

Despite these advances, it is worth noting that most computational studies remain somewhat idealized. Surface heterogeneity, competitive adsorption, and real wastewater complexity are often not fully captured, which limits direct quantitative prediction.

## 11. Computational approaches in electrochemical recovery

The application of computational methods in electrochemical systems is in many respects, more complex. Here, the focus shifts from static adsorption to dynamic processes involving electron transfer, interfacial energetics, and reaction pathways. DFT has been used to estimate reduction potentials and to model the energetics of metal ion reduction on electrode surfaces. For instance, studies on gold and palladium deposition have examined how different electrode materials influence adsorption energies of intermediate species, which in turn affects nucleation and growth behavior. Another important area is the modeling of electrode–electrolyte interfaces. Implicit and explicit solvation models are often employed to approximate the effect of the aqueous environment, although capturing the full complexity of the electrical double layer remains a challenge.^149^

Computational studies have also been used to explore competing reactions, such as hydrogen evolution, which can significantly reduce current efficiency during metal recovery. By comparing reaction energetics, it becomes possible to identify conditions under which metal deposition is favored over parasitic processes. However, compared to adsorption systems, computational electrochemistry is still developing. The need to account for applied potential, solvent structure, and time-dependent behavior makes these simulations considerably more demanding. As a result, their predictive capability, while improving, is still somewhat limited in practical terms.

## 12. Comparative perspective: adsorption *vs.* electrochemical systems

A comparison of computational approaches across adsorption and electrochemical systems reveals a clear difference in maturity and applicability. In adsorption studies, computational methods are relatively well established. They are routinely used to screen functional groups, predict selectivity, and provide molecular-level explanations for experimental observations. The systems themselves are comparatively simpler, often involving static interactions that can be reasonably approximated using cluster or periodic models. Electrochemical systems, on the other hand, present a far more complex scenario. The presence of an applied potential, coupled with solvent dynamics and interfacial charge distribution, introduces multiple variables that are difficult to capture simultaneously. As a result, computational studies in this area tend to focus on simplified models, and their conclusions are often qualitative rather than predictive. That said, the gap between the two is gradually narrowing. Advances in computational electrochemistry, including constant-potential methods and improved solvation models, are beginning to provide more realistic insights into electrode processes.

From a practical standpoint, computational methods are currently more effective in guiding adsorbent design, whereas in electrochemical systems they serve primarily to interpret observed behavior and suggest trends.

## 13. Comparative analyses of hybrid electrochemical–analytical–simulation frameworks

Recent research supports an integrated framework of four modules: solid-phase adsorption for metal capture, an electrochemical system for reversible metal loading and release, analytical instruments for real-time monitoring, and multiscale computational models that link molecular interactions to reactor-scale performance. The goal is to successfully recover important metals like Au, Ag, Pd, and Rh from wastewater using as few chemicals as possible and minimizing environmental impact.

Every module has merits and cons, and the best approach to integrate them is to streamline the process. Using controlled potentials, electrodeposition, electrosorption, electrodialysis, and new reactive electroseparations, selectively attract, deposit, and release metal cations on conductive surfaces. Such procedures employ fewer chemicals. Custom electrode surfaces and redox mediators increase selectivity. Even at low concentrations, delayed ion transport to electrodes, fouling from other ions and organics, and trade-offs between current density, energy use, and product purity exist. These issues have been reduced by forced flow through structured electrodes, pulsed potentials, and integrated electrosorption–deposition systems.

Advanced sorbents such as functionalized MOFs, ion-exchange resins, thiol- and amine-modified carbons, and treated biomass attract Au(iii) and Pd(ii) under certain conditions. Some Zr-based MOFs can contain over 200 mg of Au/g and regenerate hundreds of times, suggesting their large-scale usage. Elution requires hot acidic or organic solvents, which may negate environmental improvements unless in a closed-loop system. Process control requires dependable, constant monitoring. Portable electrochemical sensors, like stripping voltammetry or microelectrode arrays, can detect sub-ppb levels and simplify voltage adaptation. Laboratory tools like ICP-MS and ICP-OES are essential for validation and speciation analysis. A powerful framework combines field-use sensors for fast feedback with periodic ICP-based verification for accuracy, traceability, and quality assurance.

Computational modeling links molecular-scale adsorption to reactor-scale performance. DFT and machine-learning interatomic potentials predict adsorption energetics and morphologies, while Monte Carlo and molecular dynamics simulations are employed to construct isotherms and selectivity profiles. These enable continuous solvers like CFD and COMSOL to estimate electrode and packed bed flow, diffusion, and reaction. Recently developed machine learning methods can produce findings virtually as precise as DFT at a cheaper cost. This allows researchers to test several sorbents and reactor designs before experiments.

Simulation is crucial before selecting materials and optimizing procedures. Collaboration between electrochemical, analytical, and simulation parts yields actual benefits such as (i) reduced chemical usage, (ii) voltage-driven desorption functions as chemical stripping, (iii) automated control, (iv) real-time sensors adjust potentials or flow rates for purity and stability, and (v) faster design. Simulation-guided screening reduces tests to the most promising configurations, saving time and money.

Comparative investigations show that this hybrid technique is more energy-efficient and selective than standalone systems in complex multi-ion settings. Most benefits come from strong chemical affinity adsorbents and electrochemical stages for on-demand stripping and high-purity metal plating. Future directions include benchmark testing for trace-level recovery in multi-ion matrices, automated closed-loop operation integrating electrochemical sensing with periodic ICP verification, simulation-guided sorbent selection, and modular reactors with interchangeable adsorbent cartridges and electrode plates for adaptable, maintainable processes.

## 14. Conclusion

Recovering precious metals such as gold (Au), silver (Ag), palladium (Pd), and rhodium (Rh) from wastewater is becoming an essential part of sustainable resource management, offering both environmental and economic benefits. Among various approaches, adsorption stands out as a powerful technique. Advanced materials like MOFs, chitosan-based polymers, synthetic resins, carbon materials, and biosorbents have shown strong potential for selectively trapping trace amounts of these valuable metals. Electrochemical technique is a cheap, adaptable, and eco-friendly way to get metals out of wastewater. Unlike conventional adsorption, which involves many processes (adsorption, desorption, and reduction), electrochemical techniques can directly reduce metal ions to their elemental forms, although complexed metals often require coupling with adsorption. Similarly, integrating multiple analytical techniques provides comprehensive insight into the physicochemical properties of adsorbents, confirms adsorption/reduction mechanisms, and supports efficient and selective metal recovery.

If researchers couple the adsorption beds with instruments that watch the process, with electrodes that set the electrical potential, plus with computer models that calculate the outcome, they learn how each metal sticks to the surface, how long the step lasts, and which settings give the highest yield. A hybrid framework that links the adsorber, the electrode, the sensor, and the model recovers more metals with fewer extra substances, costs less, and forecasts the next operating conditions better than older frameworks that rely on a single technique. The combination of adsorption, electrochemical, computational modelling, but also simulation is shaping a powerful new platform for sustainable metals recovery, the one that bridges laboratory innovation and industrial application, while advancing circular resource management.

## Author contributions

AT: methodology, data curation, initial draft writing, SS: data curation, review & editing. AS: conceptualization, investigation, supervision, review & editing. MT: conceptualization, investigation, supervision, review & editing. AH: data curation, review & editing.

## Conflicts of interest

The authors declare that they have no conflict of interest.

## Abbreviations

A×D7(TUD@AXD7): 4,4′–(((Oxalylbis(azanediyl))bis(carbonothioyl))bis(azanediyl))bis(3-hydroxy naphthalene-1-sulphonic acid)-impregnated Amberlite
D301: Anionic tertiary amine resin
HSAB: Hard and soft acid base
CS-DAMN-AO: Chitosan–diaminomaleonitrile–amidoxime
D-SPµE: Dispersive solid-phase microextraction
CTS–TBUP: Chitosan modified with tetrabutylphosphonium ionic liquid/salt
CH-TU: Thiourea-functionalized chitosan composite
PGMs: Platinum group metals
PLS: Pregnant leach solution
ZMC-MAH-TEPA: Zinc-modified Chitosan functionalized with maleic anhydride and tetraethylenepentamine
D840: Thiourea-based resin
BIM-CS: Butylphosphonium ionic liquid modified Chitosan
MAC: Magnetic modification of activated carbon
TDOS: Total density of states
CB-RS: Carbamimidothioate-based resin
CH-TETA: Triethylenetetramine cross-linked chitosan
CCTG: Carboxymethyl Chitosan–tannic acid–graphene
BuIRs: 4-Butylaniline-impregnated resins
CR: Chitosan resin
PGM: Platinum group metals
(Au(gly)_2_): Gold glycinate complex
AC-SH-80: Sulfhydryl-functionalized activated carbon
AEDC: Adsorption and electrodeposition coupling
Ag-QDs: Silver quantum dots
BSAC: Bamboo stem activated carbon
DFT: Density functional theory
EB-COF@CNTs: Ethidium-based covalent organic framework decorated on carbon nanotubes
GO/GA: Gallic acid-modified GO
MMGH: Magnetically activated MXene/GO hydrogel
MTV-BioMOF: Multivariate biological metal when combined with organic framework
NSPC: N,S co-doped hierarchical porous carbon
PBE: Perdew Burke Ernzerhof
ESP: Electrostatic potential
PEG/G/CNT: Polyethylene glycol/graphene/carbon nanotube
QECFs: Quaternary ammonium functionalized chitosan fibers
SCG: Spent coffee grounds
SMCsr-B/PUF: Supermagnetic biochar/polyurea formaldehyde nanocomposite
SPCs: S-doped porous carbons
WEEE: Electrical and electronic equipment

## Data Availability

No primary research results, software or code have been included and no new data were generated or analyzed as part of this article.
